# TRIP12 promotes HIV-1 replication and latency reactivation by stabilizing Tat via USP7-mediated deubiquitination

**DOI:** 10.1128/jvi.00396-26

**Published:** 2026-05-13

**Authors:** Hongyun Shi, Panpan Quan, Yubao Hou, Huihan Wang, Yingchao Wang, Hong Wang, Wenyan Zhang

**Affiliations:** 1 Institute of Virology and AIDS Research, Center of Infectious Diseases and Pathogen Biology, Key Laboratory of Organ Regeneration and Transplantation of the Ministry of Education, The First Hospital of Jilin University, Changchun, China; 2 Hepatobiliary Pancreatic Surgery, The First Hospital of Jilin University, Changchun, China; 3 Department of Cadre's Wards Ultrasound Diagnostics, Ultrasound Diagnostic Center, The First Hospital of Jilin University 117971, Changchun, China; The Ohio State University, Columbus, Ohio, USA

**Keywords:** HIV-1 Tat, TRIP12, USP7, viral transcription, latency reactivation

## Abstract

HIV-1 Tat functions as a key transcriptional activator that amplifies viral transcription by recruiting host transcription elongation machinery, thereby forming a self-amplifying positive-feedback loop. Thus, cellular levels and post-translational modifications of Tat directly determine the efficiency of viral replication, as well as the establishment and reactivation of proviral latency. Elucidating the regulatory network that maintains Tat protein levels is therefore important for developing HIV cure strategies. Here, we identify the E3 ubiquitin ligase TRIP12 as a novel positive regulator of Tat stability that inhibits its proteasomal degradation. TRIP12 stabilizes Tat independently of its intrinsic E3 ligase activity by enhancing the interaction between the deubiquitinase USP7 and Tat, thereby reducing K48-linked ubiquitination of Tat, a modification typically associated with protein degradation. TRIP12 knockout significantly accelerates Tat degradation due to reduced USP7-Tat interaction, resulting in the suppression of viral transcription and impaired reactivation of latent virus. Moreover, TRIP12-mediated stabilization of Tat is consistently observed among multiple clade B HIV-1 strains tested in this study. Notably, TRIP12 mRNA levels are associated with plasma viral load and disease progression in people living with HIV, suggesting a potential clinical relevance of TRIP12 in HIV-1 pathogenesis. These findings expand our understanding of host-virus interactions and provide new insights into the molecular regulation of Tat stability, offering a potential basis for developing Tat-targeted therapeutic strategies.

**IMPORTANCE:**

HIV-1 Tat is essential for viral transcription and the establishment of latency and reactivation and represents a potential target for achieving a functional cure of acquired immunodeficiency syndrome (AIDS). In this study, we identify the host E3 ubiquitin ligase TRIP12 as a positive regulator that enhances USP7-mediated K48-linked deubiquitination of Tat, thereby stabilizing Tat protein. The TRIP12-USP7-Tat regulatory axis promotes HIV-1 replication and reactivation of latent HIV-1. This work uncovers a previously unrecognized mechanism by which the host ubiquitin system regulates Tat homeostasis and highlights potential therapeutic avenues for modulating HIV-1 latency.

## INTRODUCTION

Although ART and long-lasting HIV antiretroviral therapy effectively suppress viral replication and prolong patient survival, it cannot eliminate the integrated proviral genome residing in resting memory CD4+ T cells and other immune cells, resulting in rapid viral rebound upon treatment interruption (1–4). The persistence of latent reservoirs and immune evasion mechanisms represents major barriers to achieving a functional cure and effective vaccine development (2, 3).

During the HIV-1 replication cycle, Tat serves as a pivotal regulatory protein required for efficient viral transcription and replication (5). Tat binds to the transactivation response (TAR) element located within the HIV-1 long terminal repeat (LTR) and recruits the host positive transcription elongation factor b (P-TEFb) complex, which consists of cyclin-dependent kinase 9 (CDK9) and cyclin T1. This recruitment promotes the transition of RNA polymerase II from a paused to an elongating state, thereby markedly enhancing viral gene expression (6–8). Recently, it has also been reported that Tat overexpression can overcome the bottleneck that influences the ratio of active to latent infection, and its expression levels represent a critical determinant in the establishment of HIV latency (5). Tat establishes a powerful positive feedback loop that amplifies viral transcriptional output and determines the balance between active replication and latency. Therefore, Tat-targeted therapeutic strategies hold promise as a potent adjuvant in the pursuit of achieving a functional cure for HIV-1. Accumulating evidence indicates that the stability and function of Tat are tightly regulated by various posttranslational modifications, such as acetylation, phosphorylation, and notably ubiquitination (9, 10). For instance, host factor UHRF1 suppresses HIV-1 transcription and establishment of viral latency by increasing the ubiquitination-mediated proteasomal degradation of Tat and competing with P-TEFb complex binding to Tat (10). The host long non-coding RNA (lncRNA) NRON recruits Tat to bind with CUL4B and PSMD11, thereby facilitating its polyubiquitination and subsequent degradation via the proteasomal pathway (10). Our previous study also reported that FBXO45 ubiquitinates Tat for SQSTM1/p62-mediated autophagic degradation, thereby inhibiting HIV-1 replication and sustaining viral latency (9). However, the mechanisms by which Tat stability is maintained to sustain viral replication by resisting ubiquitin-mediated degradation remain incompletely understood. Here, we employed TurboID-based proximity labeling coupled with mass spectrometry to identify host factors interacting with Tat that regulate its stability, leading to the identification of thyroid hormone receptor interactor 12 (TRIP12) as a novel E3 ubiquitin ligase involved in Tat regulation.

TRIP12 is a conserved homolog of the E6AP C terminus (HECT) domain-containing E3 ubiquitin ligase that regulates the homeostasis of multiple substrate proteins through its ubiquitin ligase activity (11–14). It plays essential roles in various biological processes, including DNA damage repair, chromatin remodeling, oxidative stress response, and cellular proliferation and differentiation (11, 15–17). Until now, there have been no direct reports linking TRIP12 to viruses. In this study, we demonstrate that TRIP12 prevents proteasomal degradation of Tat by enhancing its interaction with the deubiquitinase USP7 and reducing K48-linked ubiquitination. Through this mechanism, TRIP12 stabilizes the Tat protein, thereby promoting Tat-dependent viral transcription, replication, and latency reactivation. These findings reveal a previously unrecognized regulatory role of TRIP12 in HIV-1 infection and provide new insights into the molecular mechanisms governing Tat stability, offering potential clues for the development of novel therapeutic strategies against HIV-1.

## RESULTS

### TRIP12 interacts with and stabilizes Tat to enhance HIV-1 LTR activity

To identify host factors that interact with and regulate HIV-1 Tat, we used a biotin-based proximity labeling system (9, 18). TurboID was fused to the N terminus of Tat to enable rapid biotinylation of nearby proteins, which were enriched using streptavidin (Fig. S1A). Mass spectrometry analysis identified several E3 ubiquitin ligases enriched in the TurboID-Tat group compared with the control (Fig. S1B). Literature review revealed that RNF20, RNF40, RNF2, UHRF1, TRIM33, and UBR7 have been reported to be associated with HIV-1 (19–24). ZNF598 was identified in another study from our group as a regulator of HIV-1. To further explore potential candidates, we predicted the interaction between Tat and TRIP12 or RNF169 using AlphaFold3 (Fig. S1C and D). The predicted structure suggested a stronger interaction interface between TRIP12 and Tat compared with RNF169; hence, we selected TRIP12 as the primary focus of our study. To validate this finding, we examined the interaction between TRIP12 and Tat in HEK293T cells. Reciprocal co-IP assays further validated the physical association between TRIP12 and Tat (Fig. S2A and B). To determine whether this interaction also occurs under physiological conditions, we conducted endogenous co-IP in HIV-1-infected Jurkat cells using an anti-TRIP12 antibody. Tat was readily detected in the TRIP12 immunocomplex, demonstrating that endogenous TRIP12 associates with Tat in infected cells (Fig. SA2C). Consistent with these results, confocal microscopy confirmed the colocalization of Tat and TRIP12 in the nuclei of HeLa cells (Fig. S2D).

**Fig 1:**
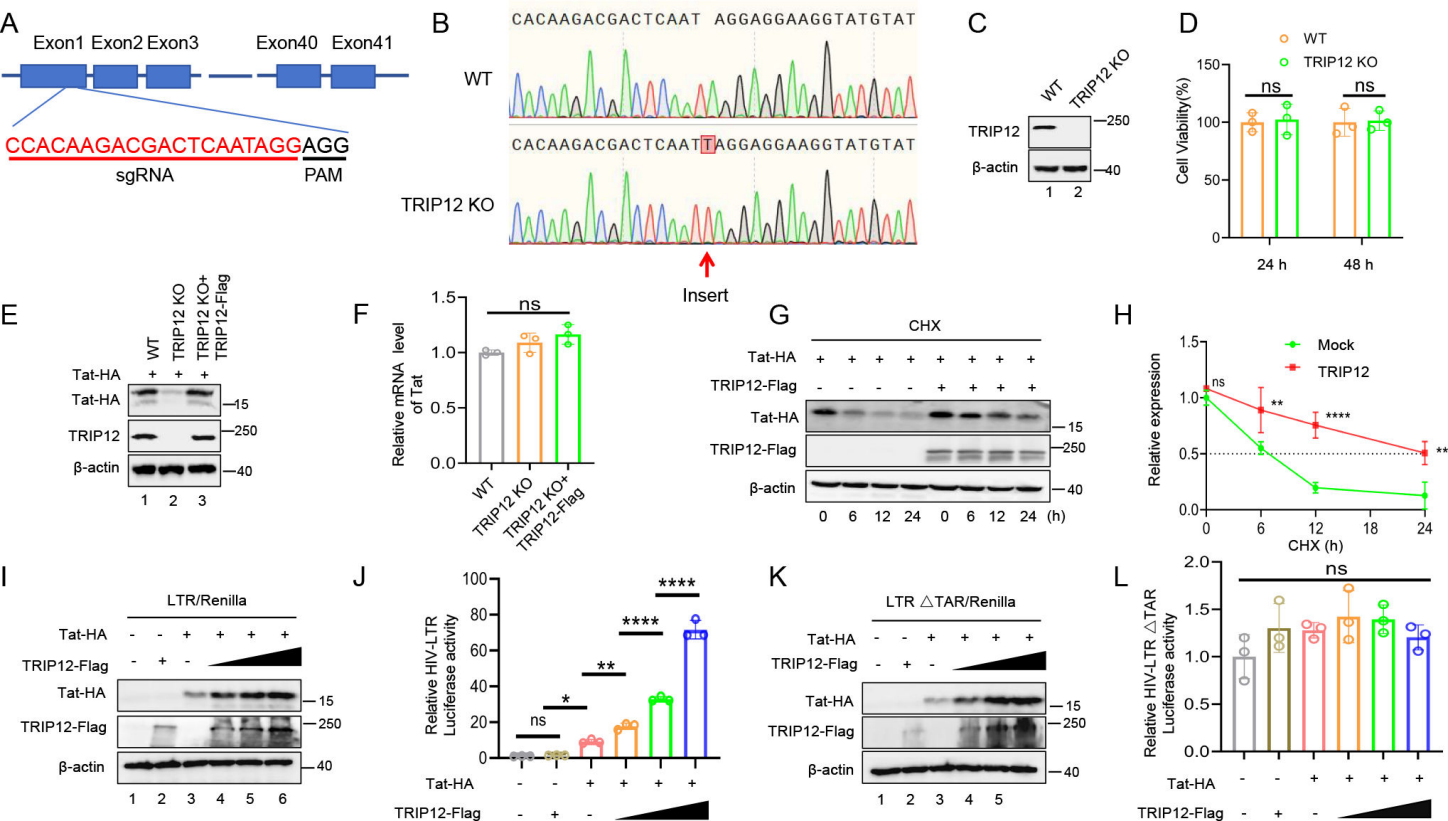
TRIP12 stabilizes Tat protein levels and enhances HIV-1 LTR activity. (**A**) Schematic representation of sgRNA targeting the TRIP12 gene. (**B**) Sanger sequencing confirms the insertion mutation in TRIP12 KO cells. (**C**) Immunoblot showing the absence of TRIP12 protein in KO cells. (**D**) Cell viability assay of WT and TRIP12 KO HEK293T cells at 24 h and 48 h. (**E**) Immunoblot analysis showing that TRIP12 deficiency decreases Tat protein levels, which are restored by re-expression of TRIP12. (**F**) RT-qPCR analysis showing that TRIP12 does not affect Tat mRNA levels. (**G and H**) TRIP12 stabilizes Tat expression. HEK293T cells were co-transfected with Tat and TRIP12 for 24 h and then treated with cycloheximide (50 μg/mL). Cell lysates were harvested at 0, 6, 12, and 24 h post-treatment. Tat stability was assessed by IB (**G**) and quantified using ImageJ (**H**). To facilitate comparison, Tat signals at 0 h were normalized across groups. (**I and J**) TRIP12 promotes Tat-mediated activation of the HIV-1 LTR. HEK293T cells were co-transfected with HIV-1 LTR-luciferase, pRenilla, Tat, and TRIP12 plasmids. After 48 h, cell lysates were analyzed by IB (**I**), and LTR-driven luciferase activity was determined using a dual-luciferase assay (**J**). (**K and L**) TRIP12 does not influence transcription driven by the HIV-1 LTR lacking the TAR element (ΔTAR). HEK293T cells were co-transfected with LTR-ΔTAR-luciferase, pRenilla, Tat, and TRIP12 plasmids. Luciferase activity was measured 48 h post-transfection using the same assay (**L**). Data are expressed as means ± SDs from three independent experiments. *P* values were calculated using a two-tailed unpaired Student’s *t*-test at each time point (D), one-way ANOVA (F, J, and L), or two-way ANOVA (H). Significance levels: *P < 0.05, **P < 0.01, ****P < 0.0001, ns (not significant).

To investigate the functional role of TRIP12, we generated TRIP12-knockout (KO) HEK293T cells using CRISPR-Cas9. Gene disruption was validated by sequencing and confirmed by immunoblotting, showing near-complete loss of TRIP12 protein without affecting cell viability (Fig. 1A through D). To further validate the functional consequence of TRIP12 depletion in this cellular context, we examined PARP1, a reported endogenous substrate of TRIP12 (25), and observed the expected increase in PARP1 abundance in TRIP12 KO cells (Fig. S2E). Loss of TRIP12 reduced Tat protein levels, which were restored by re-expression of TRIP12 (Fig. 1E). TRIP12 depletion did not affect Tat mRNA expression (Fig. 1F). After normalizing the initial Tat protein levels between the two groups, cycloheximide (CHX) chase assays showed that TRIP12 markedly prolonged the half-life of Tat compared with the control (Fig. 1G and H), indicating that TRIP12 stabilizes Tat at the protein level. Given the key role of Tat in HIV-1 transcription, we next examined whether TRIP12 modulates viral transcription. Co-transfection of HEK293T cells with HIV-1 LTR luciferase reporter, Tat, and increasing amounts of TRIP12 revealed that TRIP12 enhanced LTR-driven transcription in a Tat-dependent and dose-dependent manner (Fig. 1I and J), whereas TRIP12 alone had no effect (Fig. 1J, lane 2). Tat activates HIV-1 transcription by binding to the TAR element of the LTR. When the TAR-deficient mutant reporter was used, TRIP12 failed to enhance LTR activity through Tat (Fig. 1K and L). Together, these results demonstrate that TRIP12 enhances HIV-1 LTR activity by binding to and stabilizing Tat. To assess whether TRIP12 specifically stabilizes Tat, we co-transfected TRIP12 with several HIV-1 proteins. TRIP12 did not alter the expression of these proteins except for Tat (Fig. S2F), indicating that TRIP12 selectively stabilizes Tat.

### TRIP12 promotes HIV-1 replication by enhancing Tat-dependent transcription

Given the essential role of Tat in HIV-1 transcription and virion production, and our finding that TRIP12 stabilizes Tat, we next investigated whether TRIP12 regulates HIV-1 replication. To first assess the role of endogenous TRIP12, we examined HIV-1 replication in TRIP12 knockout HEK293T cells. Deletion of TRIP12 markedly reduced intracellular Gag-p55 and endogenous Tat protein levels, accompanied by decreased CAp24 release and infectious viral yield. These defects were restored upon re-expression of TRIP12 (Fig. S3A and B). Consistently, TRIP12 knockout significantly reduced HIV-1 Gag mRNA levels (Fig. S3C). We next evaluated whether increasing TRIP12 expression enhances viral replication. Co-transfection of the HIV-1 NL4-3 infectious clone with increasing amounts of TRIP12 in HEK293T cells resulted in a dose-dependent elevation of intracellular Gag-p55 and Tat protein levels, along with increased CAp24 release and infectious viral yield (Fig. S3D and E). TRIP12 overexpression also enhanced HIV-1 Gag mRNA expression in a dose-dependent manner (Fig. S3F) and increased viral transcripts corresponding to initiation, promoter-proximal, intermediate, and distal elongation regions (Fig. S3G). To determine whether TRIP12 promotes HIV-1 replication in a Tat-dependent manner, we examined a Tat-deficient clone (pNL4-3ΔTat). In the absence of Tat, TRIP12 overexpression did not alter Gag-p55 or CAp24 protein levels, nor did it affect Gag mRNA expression (Fig. S3H and I), indicating that TRIP12 enhances HIV-1 replication and transcription in a Tat-dependent manner. To assess whether TRIP12-mediated regulation of Tat is conserved among different HIV-1 strains, we first compared the amino acid sequences of Tat proteins derived from NL4-3, Yu2, AD8, and 89.6. Sequence alignment revealed that Tat is highly conserved across these viral strains (Fig. S4A), suggesting that TRIP12-dependent regulation of Tat may be broadly applicable. We next examined whether TRIP12 stabilizes Tat proteins from these strains. TRIP12 increased Tat protein levels across all tested viruses (Fig. S4B). Consistently, TRIP12 overexpression elevated Gag-p55 and CAp24 levels and enhanced infectious virus yield from CCR5-tropic (Yu2 and AD8), R5 X 4-tropic (89.6), and CXCR4-tropic (NL4-3) HIV-1 strains (Fig. S4C and D), indicating that TRIP12 promotes replication across multiple clade B HIV-1 strains with distinct coreceptor tropisms. Together, these results suggest that TRIP12-mediated stabilization of Tat is conserved among the clade B HIV-1 strains tested here, whereas whether this effect extends to other HIV-1 subtypes remains to be determined.

**Fig 2:**
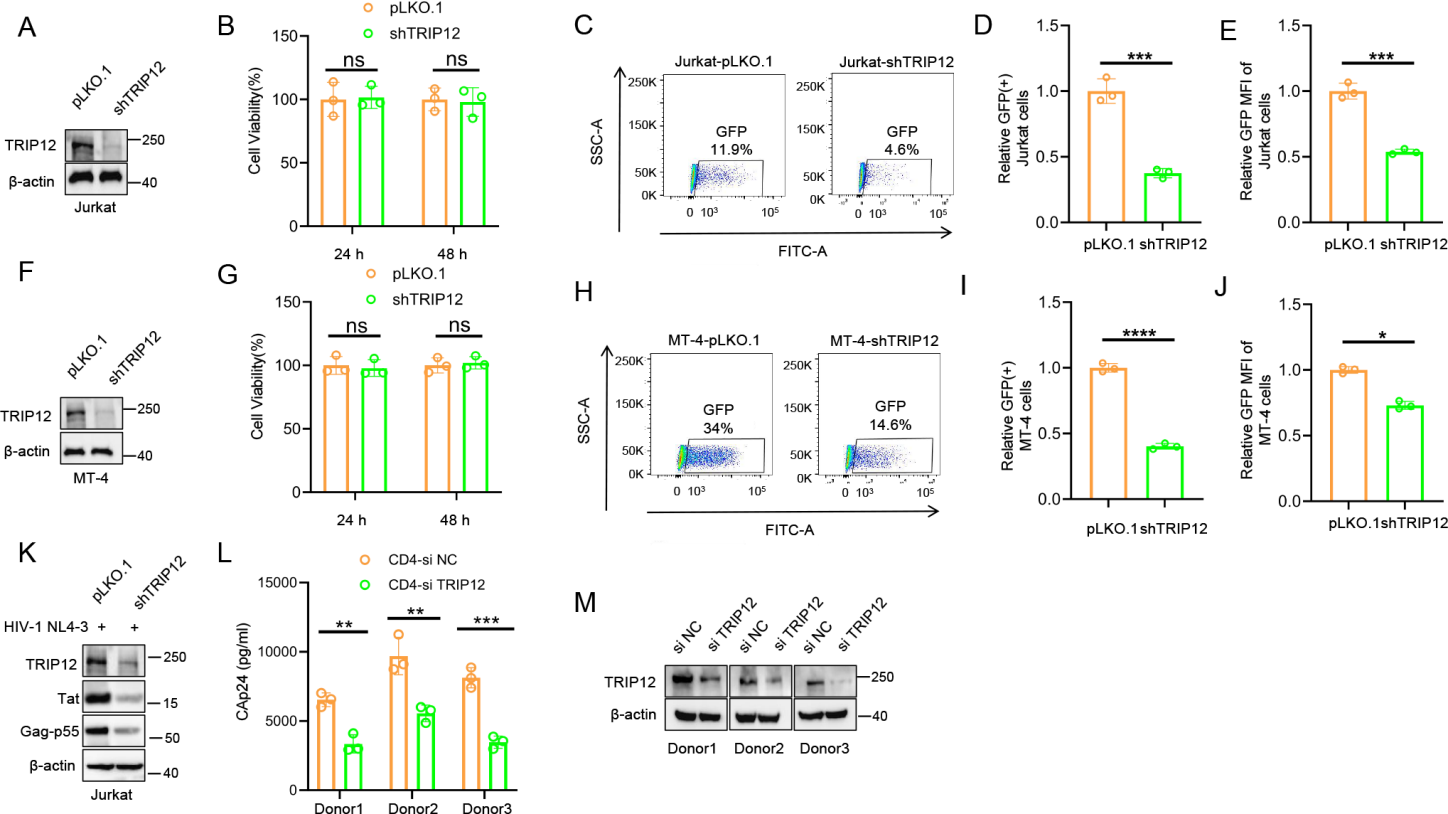
TRIP12 promotes HIV-1 replication in T cells. (**A–J**) TRIP12 knockdown suppresses HIV-1 replication in Jurkat and MT-4 cells. Jurkat and MT-4 cells stably expressing pLKO.1 or shTRIP12 were infected with VSV-G-pseudotyped pNL4-3-ΔEnv-EGFP viral particles produced in HEK293T cells. At 4 h post-infection (hpi), unbound viruses were removed by washing three times with PBS. HIV-1 replication was evaluated by flow cytometric analysis of GFP expression. TRIP12 knockdown efficiency was verified by IB in Jurkat and MT-4 cells (**A, F**). Cell viability was assessed at 24 h and 48 h (**B, G**). Representative flow cytometry plots showing GFP-positive populations are presented (**C, H**). The percentages (**D, I**) and mean fluorescence intensities (**E, J**) of GFP-positive cells were quantified. (**K**) TRIP12 stabilizes endogenous Tat protein in HIV-1-infected T cells. Jurkat-pLKO.1 or Jurkat-shTRIP12 cells were infected with HIV-1 NL4-3 for 48 h, and protein levels of TRIP12, Tat, and Gag-p55 were analyzed by IB. (**L and M**) TRIP12 depletion inhibits HIV-1 replication in primary CD4+ T cells. CD4+ T cells from three healthy donors were nucleofected with siNC or siTRIP12 and infected with HIV-1 NL4-3. At 48 h post-infection, CAp24 antigen levels in the supernatant were measured using a p24 ELISA kit (**L**), and TRIP12 knockdown efficiency was confirmed by IB (**M**). Data are presented as means ± SDs from three independent experiments. *P* values were calculated using two-tailed unpaired Student’s *t*-test (B, D, E, G, I, J, and L). Significance: *P < 0.05, **P < 0.01, ***P < 0.001, ****P < 0.0001, ns (not significant).

We further evaluated whether TRIP12 promotes HIV-1 replication in T cells. Stable TRIP12 knockdown in Jurkat and MT-4 cells (Fig. 2A, B, F, and G) significantly reduced viral gene expression following infection with the single-round reporter virus pNL4-3-ΔEnv-EGFP, as reflected by decreased percentages and mean fluorescence intensities of GFP-positive cells (Fig. 2C, D, E, H, I, and J). To assess the impact of TRIP12 on replication-competent virus, Jurkat cells were infected with wild-type HIV-1 NL4-3. Under these multi-round infection conditions, TRIP12 silencing markedly decreased endogenous Tat and Gag-p55 protein levels (Fig. 2K), indicating that TRIP12 facilitates viral gene expression and replication during productive infection. To further validate this observation in primary CD4+ T cells, TRIP12 was silenced by nucleofection in cells from three healthy donors, followed by infection with replication-competent HIV-1. TRIP12 knockdown markedly reduced CAp24 antigen levels in primary CD4+ T cells from three healthy donors (Fig. 2L and M). Together, these results demonstrate that TRIP12 enhances HIV-1 replication under both single-round and multi-round infection settings in T cell lines and primary CD4+ T cells.

### TRIP12 stabilizes Tat by inhibiting K48-linked ubiquitination

**Fig 3:**
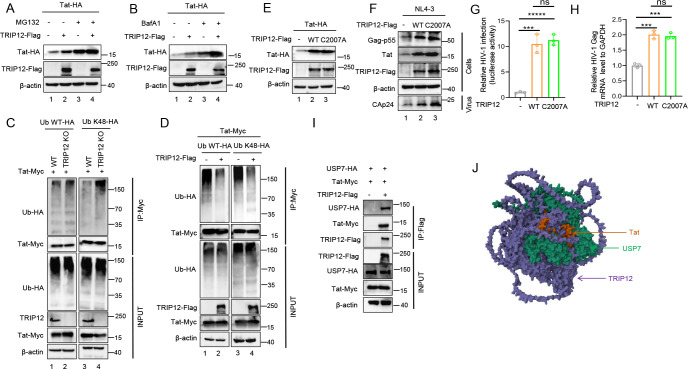
TRIP12 stabilizes Tat by inhibiting K48-linked ubiquitination, independent of its E3 ubiquitin ligase activity. (**A**) TRIP12 regulates Tat stability via the proteasomal degradation pathway. HEK293T cells were co-transfected with Tat-HA and TRIP12-Flag expression plasmids. After 36 h, the cells were treated with or without MG132 (10 μM, 8 h) and analyzed by IB. MG132 attenuated the TRIP12-dependent differences in Tat protein levels, supporting a proteasome-dependent mechanism. (**B**) TRIP12 does not regulate Tat through the lysosomal pathway. HEK293T cells were treated with or without BafA1 (10 nM, 8 h) after co-transfection as in panel A, followed by IB. TRIP12 increased Tat levels irrespective of BafA1 treatment. (**C and D**) TRIP12 counteracts K48-linked ubiquitination of Tat. HEK293T cells were co-transfected with Tat-Myc, HA-Ub (WT or K48), and TRIP12-Flag as indicated. Lysates were subjected to IP with anti-Myc and IB with anti-HA. TRIP12 knockout increased, whereas TRIP12 expression reduced, the K48-linked ubiquitination of Tat. (**E–H**) The catalytic activity of TRIP12 is dispensable for Tat stabilization and HIV-1 replication. Cells were transfected with wild-type (WT) or catalytic mutant (C2007A) TRIP12 together with Tat-HA or pNL4-3. Tat and Gag-p55/CAp24 were assessed by IB (**E and F**); infectious HIV-1 production was quantified in TZM-bl cells (**G**); HIV-1 Gag mRNA was measured by RT-qPCR (**H**). (**I**) TRIP12 interacts with USP7 and Tat. Co-IP of Tat-Myc with TRIP12-Flag and USP7-HA is shown. (**J**) AlphaFold3-predicted structural model of the Tat-TRIP12-USP7 complex. Prior to cell harvest, cells were treated with 10 µM MG132 for 8 h to ensure that Tat protein levels were comparable across all samples. This approach allowed accurate assessment of the interactions between target factors in subsequent co-IP experiments without interference from variations in input levels (C, D, and I). Data are shown as means ± SDs from three independent experiments. *P* values were calculated using one-way ANOVA (G and H). Significance: ***P < 0.001, ****P < 0.0001, ns (not significant).

Previous studies have shown that the HIV-1 Tat protein undergoes degradation through either the proteasomal pathway (10, 26, 27) or the autophagy-lysosomal pathway (9, 28). To determine how TRIP12 stabilizes Tat, HEK293T cells were transfected with Tat and TRIP12, followed by treatment with the proteasome inhibitor MG132 or the autophagy inhibitor bafilomycin A1 (BafA1), with DMSO serving as the negative control. As shown in Fig. 3A, MG132 abolished the stabilizing effect of TRIP12 on Tat, resulting in comparable Tat levels between control and TRIP12-expressing cells, whereas BafA1 treatment had no such effect (Fig. 3B). These findings indicate that TRIP12 likely stabilizes Tat through a proteasome-dependent pathway rather than an autophagic-lysosomal mechanism. Proteasomal degradation of Tat generally requires K48-linked ubiquitination (27, 29, 30). We therefore examined whether TRIP12 affects overall or K48-linked ubiquitination of Tat. As shown in Fig. 3C, knockout of endogenous TRIP12 markedly increased total and K48-linked ubiquitination of Tat, whereas overexpression of TRIP12 had the opposite effect, leading to reduced ubiquitination levels (Fig. 3D). Given that TRIP12 is a HECT-type E3 ubiquitin ligase, we next tested whether its catalytic activity contributes to this regulation. A catalytically inactive mutant, TRIP12 C2007A, was generated and compared with wild-type TRIP12. To verify that the C2007A mutant was defective in its canonical E3 ligase activity in this cellular context, we examined PARP1, a reported endogenous target of TRIP12 (25), and found that wild-type TRIP12, but not the C2007A mutant, reduced PARP1 abundance as expected (Fig. S4E). Both wild-type and C2007A mutants increased Tat protein expression and enhanced HIV-1 replication and transcription to a similar extent (Fig. 3E through H), indicating that TRIP12 functions independently of its E3 ligase activity.

Since TRIP12 reduces K48-linked ubiquitination of Tat without requiring its own catalytic activity, we hypothesized that it may act through an intermediate factor. A PubMed search using the keyword “TRIP12” identified 119 related publications, among which 10 reported a close association between TRIP12 and the deubiquitinase USP7, suggesting that USP7 can recruit TRIP12 to promote substrate degradation (31–33). Although no prior study has shown TRIP12 recruiting USP7 to stabilize target proteins, we hypothesized that TRIP12 may instead recruit USP7 to deubiquitinate and stabilize Tat. Consistent with this possibility, previous work demonstrated that USP7 stabilizes HIV-1 Tat by reducing its K48-linked ubiquitination, thereby promoting viral replication (34). To verify the potential relationship among TRIP12, USP7, and Tat, we performed co-IP and confocal microscopy analyses. Co-IP assays demonstrated the interaction among TRIP12, USP7, and Tat (Fig. 3I), and confocal microscopy revealed that TRIP12, USP7, and Tat co-localize in the nucleus of HeLa cells (Fig. S4F). Moreover, AlphaFold3-based structural prediction suggested the existence of a ternary complex formed by TRIP12, USP7, and Tat (Fig. 3J). Together, these results suggest that TRIP12 may stabilize Tat by recruiting USP7 to reduce its K48-linked ubiquitination, thereby promoting HIV-1 replication.

### TRIP12 enhances USP7-Tat interaction to promote Tat stability and HIV-1 replication

**Fig 4:**
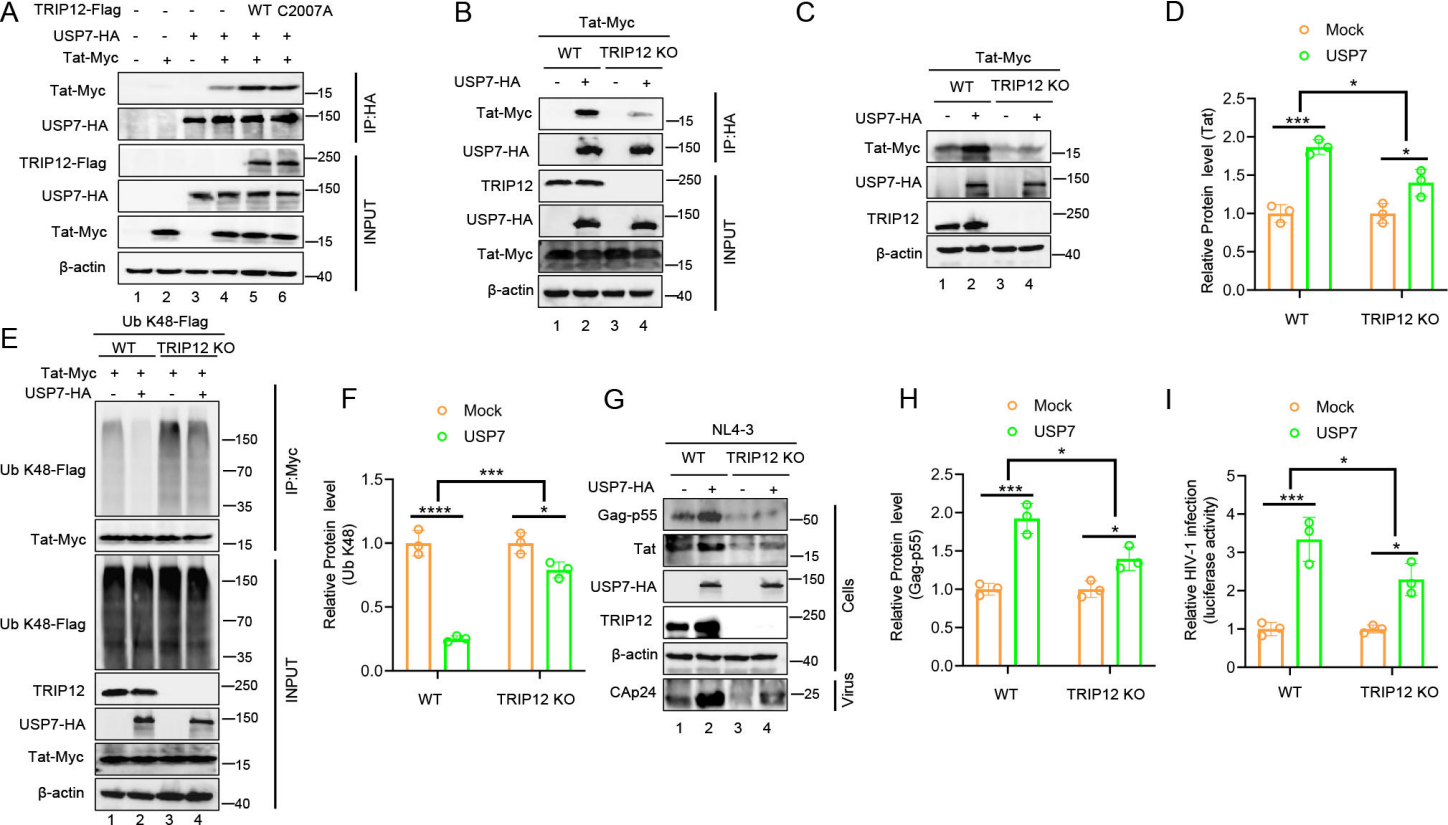
TRIP12 enhances USP7-Tat interaction to promote Tat stability and HIV-1 replication. (**A**) TRIP12 promotes the interaction between USP7 and Tat. HEK293T cells were co-transfected with the indicated plasmids expressing Tat-Myc, USP7-HA, and either wild-type (WT) or catalytic mutant (C2007A) TRIP12-Flag. Cell lysates were subjected to IP with anti-HA and analyzed by IB using the indicated antibodies. (**B**) TRIP12 knockout weakens the interaction between USP7 and Tat. TRIP12 WT and KO HEK293T cells were co-transfected with USP7-HA and Tat-Myc. After 48 h, cell lysates were immunoprecipitated with anti-HA antibody and analyzed by IB using the indicated antibodies. (**C and D**) TRIP12 knockout decreases USP7-mediated stabilization of Tat. TRIP12 WT and KO HEK293T cells were co-transfected with USP7-HA and Tat-Myc. After 48 h, cell lysates were analyzed by IB to detect Tat and the indicated proteins (**C**), and Tat protein levels were quantified using ImageJ software (**D**). (**E and F**) TRIP12 knockout attenuates the ability of USP7 to reduce K48-linked ubiquitination of Tat. TRIP12 WT and KO HEK293T cells were co-transfected with Tat-Myc, USP7-HA, and Flag-tagged K48-only ubiquitin plasmids. Cell lysates were immunoprecipitated with anti-Myc antibody and analyzed by IB using the indicated antibodies (**E**). The levels of K48-linked ubiquitination were quantified using ImageJ software (**F**). (**G–I**) TRIP12 knockout impairs. USP7-mediated HIV-1 replication. TRIP12 WT and KO HEK293T cells were co-transfected with pNL4-3 and USP7-HA plasmids. Cell lysates were analyzed by IB to detect Gag-p55, Tat, and CAp24 proteins (**G**). Gag-p55 levels were quantified by ImageJ (**H**), and infectious HIV-1 production was measured in TZM-bl cells using a luciferase activity assay (**I**). Prior to cell harvest, cells were treated with 10 µM MG132 for 8 h to ensure that Tat protein levels were comparable across all samples. This approach allowed accurate assessment of the interactions between target factors in subsequent co-IP experiments without interference from variations in input levels (A, B, and E). Data are expressed as means ± SDs from three independent experiments. *P* values were calculated using two-way ANOVA (D, F, H, and I). Significance: *P < 0.05, ***P < 0.001, ****P < 0.0001.

To delineate how TRIP12 leverages USP7 to act on Tat, we performed co-IP in HEK293T cells co-expressing Tat, USP7, and either TRIP12-WT or the C2007 mutant. Both TRIP12-WT and C2007 markedly enhanced the USP7-Tat association (Fig. 4A), consistent with the ability of both forms to increase Tat levels and HIV-1 replication shown in Fig. 3E through H. In TRIP12 KO cells, the USP7-Tat interaction was substantially reduced (Fig. 4B). Functionally, loss of TRIP12 blunted the capacity of USP7 to stabilize Tat (Fig. 4C and D) and to diminish K48-linked ubiquitination of Tat (Fig. 4E and F). Consequently, USP7 failed to boost Gag-p55 expression, CAp24 release, and infectious virus yield when TRIP12 was absent (Fig. 4G through I).

**Fig 5:**
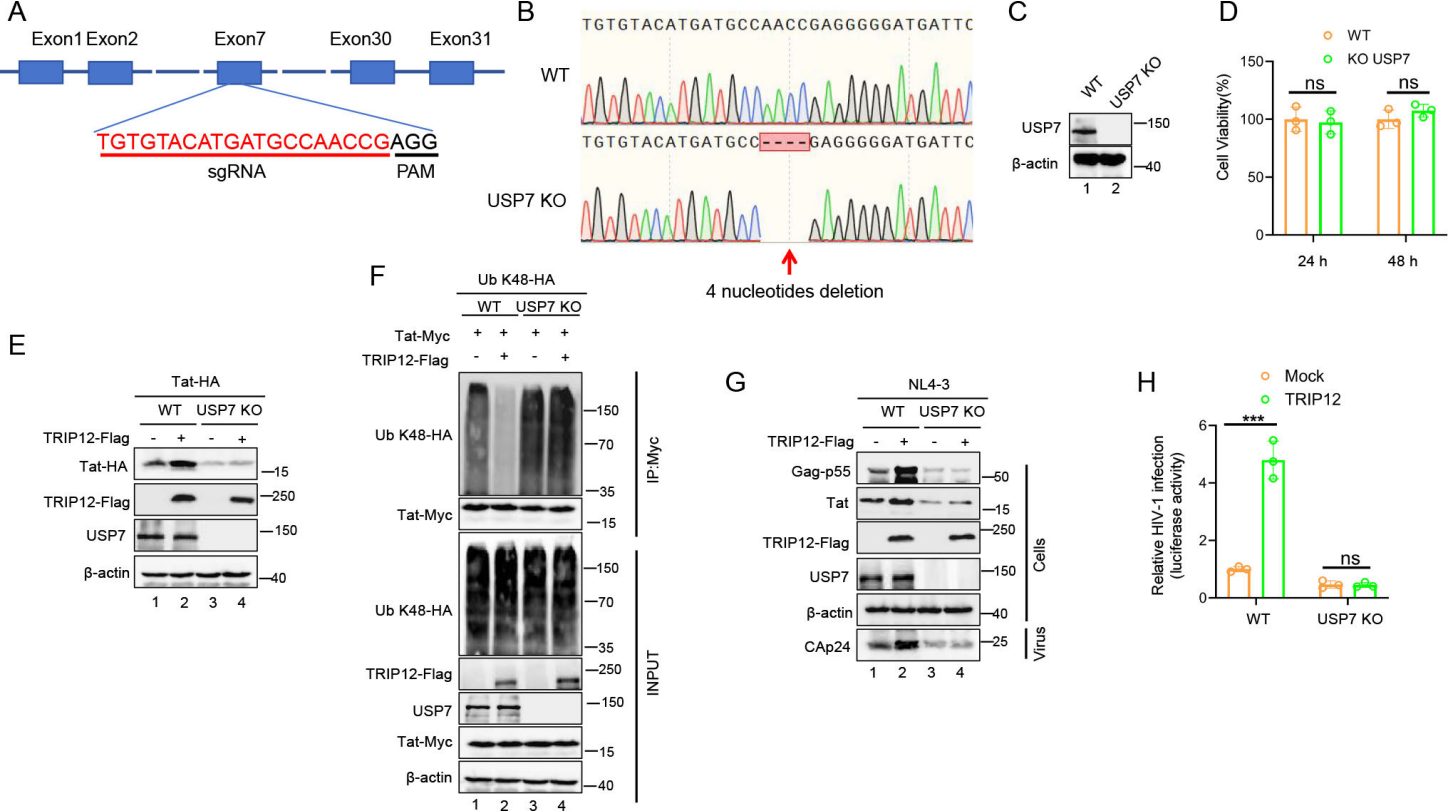
TRIP12-mediated Tat regulation depends on USP7. (**A and B**) Schematic diagram of the sgRNA targeting site within the USP7 gene (**A**) and Sanger sequencing confirmation of a four-nucleotide deletion in the USP7 knockout (KO) HEK293T cell line (**B**). (**C**) IB verification of USP7 knockout efficiency in HEK293T cells. (**D**) Cell viability of WT and USP7 KO HEK293T cells at 24 h and 48 h was assessed by CCK-8 assay. (**E**) USP7 knockout markedly impairs TRIP12-mediated Tat stabilization. WT or USP7 KO HEK293T cells were co-transfected with Tat-HA and TRIP12-Flag plasmids, followed by IB analysis with the indicated antibodies. (**F**) USP7 knockout substantially reduces the ability of TRIP12 to reduce K48-linked ubiquitination of Tat. WT or USP7 KO HEK293T cells were co-transfected with Tat-Myc, HA-tagged K48-only ubiquitin, and TRIP12-Flag plasmids. Cell lysates were immunoprecipitated with anti-Myc and analyzed by IB using the indicated antibodies. (**G and H**) USP7 knockout largely abolishes the TRIP12-mediated enhancement of HIV-1 replication. WT or USP7 KO HEK293T cells were co-transfected with pNL4-3 and TRIP12-Flag plasmids. Cell lysates were analyzed by IB to detect Gag-p55, Tat, and CAp24 proteins (**G**), and infectious HIV-1 production was quantified in TZM-bl cells using a luciferase activity assay (**H**). Prior to cell harvest, cells were treated with 10 µM MG132 for 8 h to ensure that Tat protein levels were comparable across all samples. This approach allowed accurate assessment of the interactions between target factors in subsequent co-IP experiments without interference from variations in input levels (**F**). Consistent with previous reports, USP7 knockout reduced TRIP12 protein levels. Therefore, TRIP12 expression was adjusted to comparable levels between groups for mechanistic analyses (E, F, and G). Data are shown as means ± SDs from three independent experiments. *P* values were calculated using a two-tailed unpaired Student’s *t*-test (D and H). Significance: ***P < 0.001, ns (not significant).

To further determine whether TRIP12-mediated regulation of Tat depends on USP7, we generated USP7 KO HEK293T cells using CRISPR-Cas9 genome editing. Gene disruption was validated by sequencing and confirmed by immunoblot analysis, demonstrating near-complete loss of USP7 protein without affecting cell viability (Fig. 5A through D). As an additional functional validation of USP7 knockout, we examined MDM2, a well-established endogenous target regulated by USP7 (35), and observed the expected reduction in MDM2 protein levels in USP7 KO cells (Fig. S4G). In USP7 KO cells, TRIP12 failed to stabilize Tat protein (Fig. 5E). As USP7 itself removes K48-linked ubiquitin chains from Tat to stabilize it, the overall Tat level was lower in the USP7 KO group than in the control group. Consistently, TRIP12 lost its ability to reduce K48-linked ubiquitination of Tat (Fig. 5F). Co-transfection of the pNL4-3 with TRIP12 in USP7 KO HEK293T cells failed to increase intracellular Gag-p55 and Tat protein levels or enhance CAp24 release and infectious virus yield (Fig. 5G and H). To further evaluate the requirement of USP7 enzymatic activity, we treated cells with the USP7 catalytic inhibitor GNE-6640 (36, 37). We first identified a functionally effective concentration of GNE-6640 that reduced Tat abundance without affecting cell viability (Fig. S5A and B). Under these conditions, GNE-6640 markedly impaired the ability of TRIP12 to enhance Tat stability (Fig. S5C) and infectious HIV-1 yield (Fig. S5D and E). These findings are consistent with the genetic ablation data in USP7 knockout cells and support the conclusion that TRIP12 regulates Tat stability and HIV-1 replication in a USP7 activity-dependent manner. Notably, USP7 has been reported to stabilize TRIP12 expression (31). Therefore, when comparing USP7 knockout and wild-type cells, as well as cells treated with or without GNE-6640, TRIP12 expression levels were adjusted to comparable levels prior to assessing its effect on Tat stability. This normalization ensured that any observed changes in Tat were not secondary to alterations in TRIP12 abundance. Additionally, gradient co-transfection of TRIP12 and USP7 in HEK293T cells showed that TRIP12 did not affect USP7 protein stability (Fig. S5F), indicating that the regulatory relationship is not reciprocal under these conditions. Together, TRIP12 strengthens USP7-Tat binding, thereby lowering Tat K48-linked ubiquitination, preventing proteasomal degradation, stabilizing Tat, and promoting HIV-1 transcription and replication.

### TRIP12 promotes latent HIV-1 reactivation

**Fig 6:**
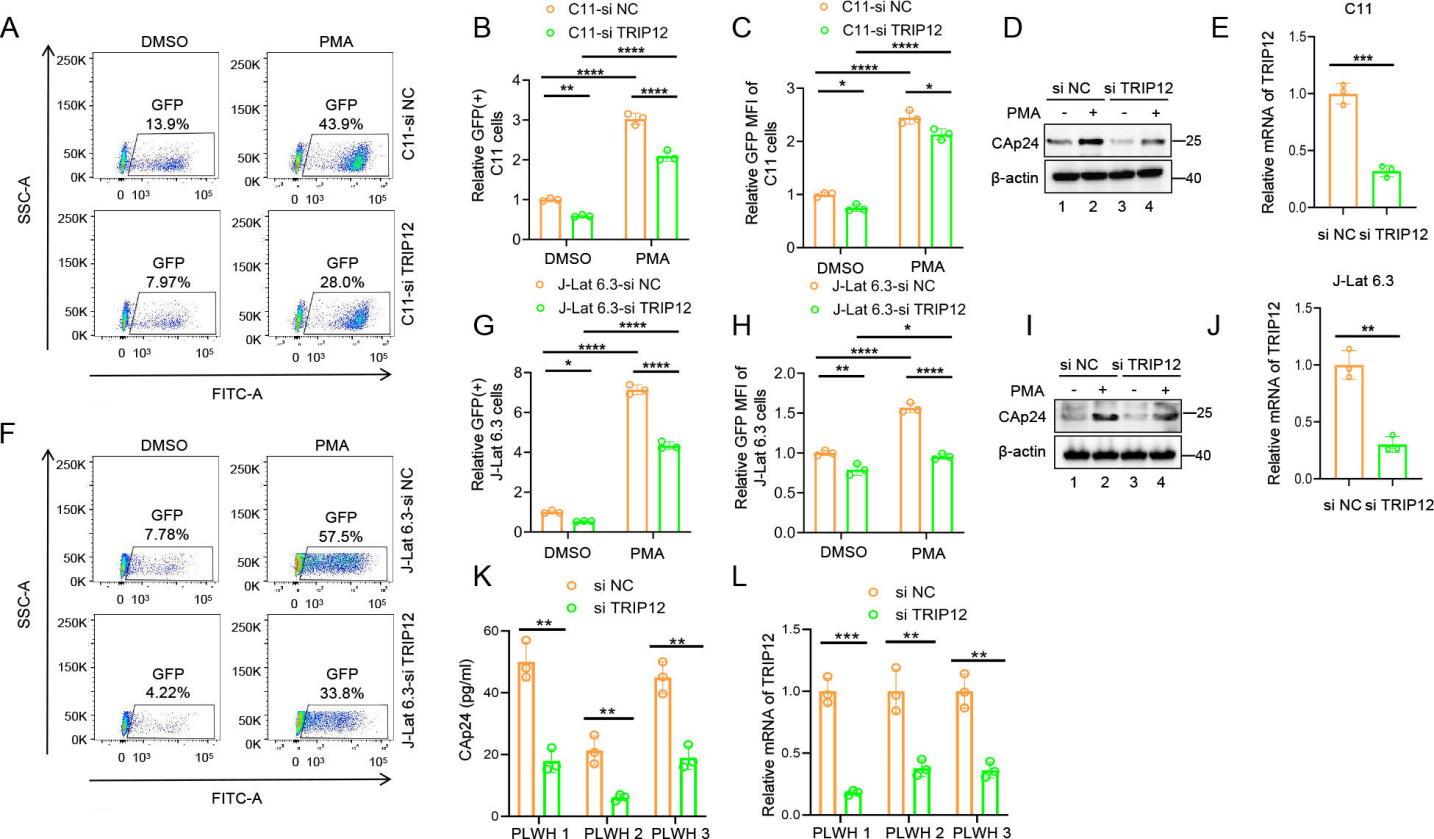
TRIP12 promotes latent HIV-1 reactivation. (**A–E**) TRIP12 knockdown attenuates PMA-induced HIV-1 reactivation in C11 cells. C11 cells were transfected with siNC or siTRIP12 and stimulated with PMA (20 nM) or DMSO for 48 h. HIV-1 reactivation was assessed by flow cytometric analysis of GFP expression. Representative flow cytometry plots are shown (**A**). The percentage of GFP-positive cells (**B**) and the mean fluorescence intensity (MFI) (**C**) were quantified. The levels of CAp24 were examined by IB (**D**). TRIP12 mRNA levels were assessed by RT-qPCR and normalized to GAPDH relative to siNC (**E**). (**F–J**) TRIP12 depletion suppresses PMA-induced HIV-1 reactivation in J-Lat 6.3 cells. J-Lat 6.3 cells were transfected with siNC or siTRIP12 and stimulated with PMA (20 nM) or DMSO for 48 h. GFP expression was analyzed by flow cytometry (**F**), and the percentage of GFP-positive cells (**G**) and the GFP MFI (**H**) were quantified. CAp24 levels were determined by IB (**I**). TRIP12 mRNA levels were assessed by RT-qPCR and normalized to GAPDH relative to siNC (**J**). (**K**) Knockdown of TRIP12 impaired HIV-1 reactivation in primary CD4+ T cells. CD4+ T cells isolated from HAART-treated people living with HIV (PLWH) (*n* = 3) were nucleofected with TRIP12-specific siRNA. Following stimulation with PHA-M (5 µg/mL) in the presence of IL-2 (50 U/mL), viral reactivation was evaluated by measuring p24 antigen levels in the culture supernatant using ELISA. (**L**) The efficiency of TRIP12 silencing was verified by quantitative RT-qPCR, with mRNA levels normalized to GAPDH and expressed relative to siNC. Data are shown as means ± SDs from three independent experiments. *P* values were calculated using two-tailed unpaired Student’s *t*-test (E, J, K, and L) or two-way ANOVA (B, C, G, and H). Significance: *P < 0.05, **P < 0.01, ***P < 0.001, ****P < 0.0001.

Given the critical role of Tat in transcriptional elongation and reactivation of latent HIV-1 (38, 39), we hypothesized that TRIP12 may promote HIV-1 reactivation by stabilizing Tat. To test this, siRNA targeting TRIP12 was nucleofected into two latently infected HIV-1 cell models, C11 and J-Lat 6.3, to knock down TRIP12 expression. Cells were then treated with either DMSO or the T cell activator PMA, followed by flow cytometric analysis (Fig. 6A and F). The proportion of GFP-positive cells was markedly reduced upon TRIP12 knockdown in both models, indicating decreased HIV-1 reactivation (Fig. 6B and G). Quantification of the mean fluorescence intensity (MFI) of GFP showed consistent results, further confirming that TRIP12 knockdown reduced HIV-1 reactivation (Fig. 6C and H). Furthermore, IB revealed that CAp24 protein levels, a marker of HIV-1 gene expression, were similarly reduced (Fig. 6D and I). The efficiency of TRIP12 silencing was confirmed by RT-qPCR (Fig. 6E and J). Collectively, these findings indicate that TRIP12 facilitates reactivation of latent HIV-1 in established latency models. Consistent with these observations, silencing TRIP12 also attenuated HIV-1 reactivation in primary CD4+ T cells isolated from HAART-treated people living with HIV (*n* = 3). Following PHA-M stimulation, p24 antigen production in the culture supernatant was significantly reduced upon TRIP12 knockdown compared with siNC controls (Fig. 6K). Efficient depletion of TRIP12 mRNA was confirmed by RT-qPCR (Fig. 6L). Together, these data further support a role for TRIP12 in promoting HIV-1 reactivation in both cell line-based models and primary CD4+ T cells.

### TRIP12 expression is elevated during HIV-1 infection and reactivation

**Fig 7:**
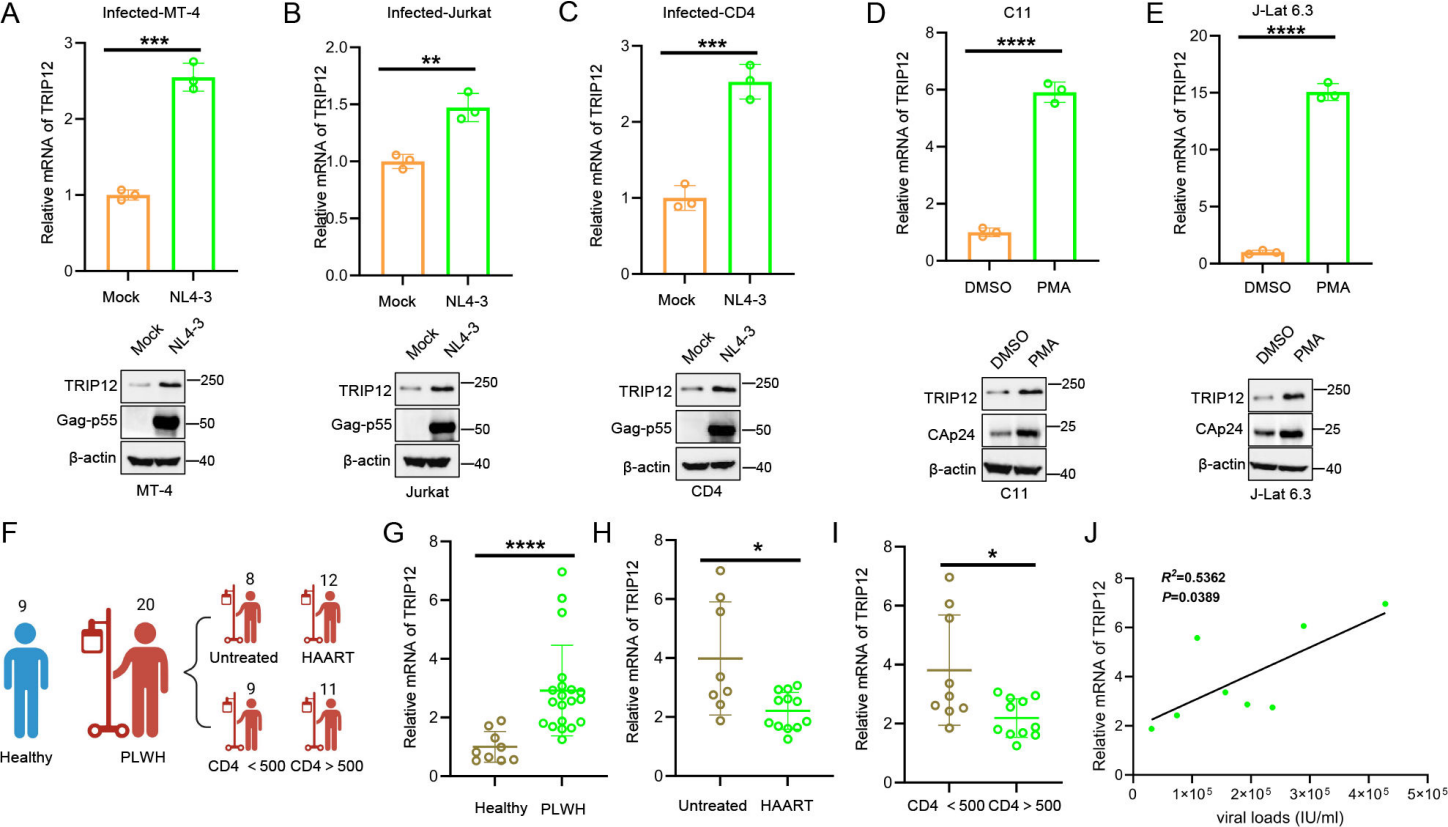
TRIP12 expression is upregulated during HIV-1 infection and reactivation. (**A-C**) TRIP12 expression is elevated upon HIV-1 infection. MT-4 (**A**), Jurkat (**B**), and primary CD4+ T cells (**C**) were infected with HIV-1 NL4-3 for 48 h. TRIP12 mRNA levels were measured by RT-qPCR, and protein levels of TRIP12 and Gag-p55 were analyzed by IB. (**D and E**) TRIP12 expression is induced during HIV-1 reactivation. Latently infected C11 (**D**) and J-Lat 6.3 (**E**) cells were treated with PMA (20 nM) or DMSO for 48 h. TRIP12 mRNA levels were quantified by RT-qPCR, and protein levels of TRIP12 and CAp24 were analyzed by IB. (**F–I**) TRIP12 expression in CD4+ T cells from people living with HIV and healthy controls. (**F**) Schematic diagram showing the study groups: 9 healthy donors and 20 people living with HIV (8 untreated, 12 HAART-treated; 9 with CD4 < 500 cells/µL, 11 with CD4 > 500 cells/µL). TRIP12 mRNA levels in CD4+ T cells were measured by RT-qPCR and compared between healthy controls and people living with HIV (**G**), between untreated people living with HIV and those receiving HAART (**H**), and between people living with HIV with low or high CD4+ T cell counts (**I**). The schematic in panel F was generated using BioRender (http://biorender.com/). (**J**) Association between TRIP12 mRNA levels and plasma viral load in untreated people living with HIV. TRIP12 expression was plotted against plasma viral load (IU/mL), and correlation was assessed using linear regression analysis (*n* = 8). Data are shown as means ± SDs from three independent experiments. *P* values were calculated using a two-tailed unpaired Student’s *t*-test (A to E), Mann-Whitney *U* test (G), or Welch’s *t*-test for unequal variances assumed (H and I). Significance: *P < 0.05, **P < 0.01, ***P < 0.001, ****P < 0.0001.

We next examined the association between TRIP12 expression and HIV-1 replication or latency reactivation. In MT-4, Jurkat, and primary CD4+ T cells, HIV-1 infection markedly increased TRIP12 mRNA levels and was accompanied by an increase in TRIP12 protein abundance (Fig. 7A through C). Similarly, upon reactivation of latent HIV-1 by the T cell activator PMA, TRIP12 expression was markedly upregulated in the latent cell lines C11 and J-Lat 6.3 (Fig. 7D and E). To further assess the clinical relevance of TRIP12, we analyzed TRIP12 mRNA levels in peripheral blood CD4+ T cells from 9 healthy donors and 20 people living with HIV, who were further stratified based on HAART treatment status and CD4+ T cell counts (Fig. 7F). TRIP12 expression was significantly higher in CD4+ T cells from people living with HIV than in those from healthy controls (Fig. 7G). Moreover, people living with HIV who were not receiving antiretroviral therapy exhibited higher TRIP12 levels than those receiving HAART (Fig. 7H). In addition, among people living with HIV, TRIP12 expression was higher in those with CD4+ T cell counts below 500 cells/µL than in those with counts above 500 cells/µL (Fig. 7I). To further evaluate the association between TRIP12 expression and viral load, we analyzed the correlation between TRIP12 mRNA levels and plasma viral load. The results revealed a positive correlation between these two parameters (Fig. 7J). These results suggest that TRIP12 expression is associated with viral load and selected clinical parameters in people living with HIV.

## DISCUSSION

HIV-1 Tat is a key regulatory factor that drives LTR-dependent transcriptional elongation by forming a positive feedback loop with host cofactors and the TAR element, thereby controlling viral gene expression, replication, and latency reactivation (40). Reduced Tat expression or impaired Tat function markedly suppresses viral transcription, making Tat a central target for functional cure strategies against HIV-1. Several Tat-directed therapeutic approaches have been developed (41, 42). Tat is a small, highly modifiable protein that undergoes multiple posttranslational modifications, including ubiquitination, acetylation, phosphorylation, and others (43, 44). Among these, ubiquitination plays a pivotal role in regulating Tat function, as distinct E3 ubiquitin ligases influence its fate through different mechanisms. For example, HDM2-mediated ubiquitination enhances Tat’s transcriptional activity (45), whereas CHIP and FBXO45 promote Tat degradation through the ubiquitin-proteasome or autophagy pathways (9, 29). Given Tat’s essential role in HIV-1 transcription, replication, and latency reactivation, and the critical impact of E3 ubiquitin ligases on Tat regulation, this study identifies TRIP12 as a novel E3 ligase that promotes HIV-1 replication and latency reactivation by stabilizing Tat.

TRIP12 was identified as a Tat-interacting protein and further confirmed to associate and colocalize with Tat in the nucleus. Functionally, TRIP12 stabilized Tat, prolonged its half-life, and enhanced Tat-dependent HIV-1 LTR activity. TRIP12 also promoted HIV-1 replication in both HEK293T cells and T cells in a Tat-dependent manner and showed similar effects across the clade B HIV-1 strains tested in this study. Mechanistically, TRIP12 stabilized Tat by reducing its K48-linked ubiquitination and proteasomal degradation. Notably, this effect was independent of the intrinsic E3 ligase activity of TRIP12 and was likely mediated through USP7. Although the close functional relationship between TRIP12 and USP7 has been reported, with USP7 known to recruit TRIP12 to degrade certain substrates (32, 33), our study is the first to demonstrate that TRIP12 can conversely recruit USP7 to stabilize its substrate proteins, thereby refining the understanding of their cooperative roles. Specifically, TRIP12 markedly enhanced the interaction between USP7 and Tat, enabling USP7 to efficiently remove K48-linked ubiquitin chains from Tat, thereby increasing Tat stability and promoting viral replication. Moreover, both USP7 knockout and treatment with the USP7 enzymatic inhibitor GNE-6640 markedly impaired the effects of TRIP12, demonstrating that TRIP12-mediated regulation of Tat stability is dependent on USP7. Notably, a study published in 2016 (46) reported that TRIP12 mediates the degradation of USP7, whereas a later study in 2022 (36) and our current findings (Fig. S5F) demonstrated that TRIP12 does not affect USP7 protein expression. This discrepancy may result from differences in cell types, experimental conditions, or cellular protein homeostasis environments. In addition, knockdown of TRIP12 significantly impaired HIV-1 latency reactivation in the C11 and J-Lat 6.3 models, as well as in primary CD4+ T cells isolated from HAART-treated individuals, indicating a critical role for TRIP12 in latency reversal. Beyond its role in Tat stabilization, TRIP12 has been implicated in several cellular processes, including DNA damage response and repair, chromatin regulation, oxidative stress response, and cell proliferation or differentiation (11, 15–17). Some of these processes may also influence HIV-1 replication and latency, particularly because viral transcription is closely linked to the host chromatin environment and cellular state. It is therefore possible that TRIP12 affects HIV-1 biology through additional indirect mechanisms beyond the USP7-Tat axis. However, based on our current data, the most directly supported role of TRIP12 in this setting is the regulation of Tat stability through USP7-dependent deubiquitination.

Given the potential clinical relevance of host factors in HIV-1 pathogenesis, we examined the association between TRIP12 expression and HIV-1 disease-related parameters. TRIP12 expression was increased following HIV-1 infection or latency reactivation. In clinical samples, people living with HIV showed higher TRIP12 mRNA levels than healthy controls, whereas those receiving antiretroviral therapy or with CD4+ T cell counts above 500 cells/µL exhibited comparatively lower TRIP12 expression. In addition, TRIP12 mRNA levels were positively correlated with plasma viral load. Together, these findings suggest that TRIP12 expression is associated with HIV-1 disease activity and latency reactivation. It has been reported that some E3 ligases, particularly several TRIM family E3 ligases, are induced by type I and type II interferons (IFNs) (47, 48). HIV infection may also trigger innate immune activation. Therefore, we cannot exclude the possibility that the increased TRIP12 expression observed in our study is, at least in part, a consequence of immune activation rather than a direct cause of higher viral burden. In addition, we did not assess TRIP12 protein levels in samples from people living with HIV, and our current data do not distinguish whether the observed increase occurs specifically in infected cells or also in bystander cells. These issues will require further investigation in future studies. At present, whether TRIP12 itself is an IFN-inducible protein remains unclear. Another limitation is that, to our knowledge, no specific small-molecule modulators directly targeting TRIP12 have been reported. Nevertheless, our findings provide a rationale for exploring TRIP12 as a potential host-directed target for regulating HIV-1 replication and latency.

**Fig 8:**
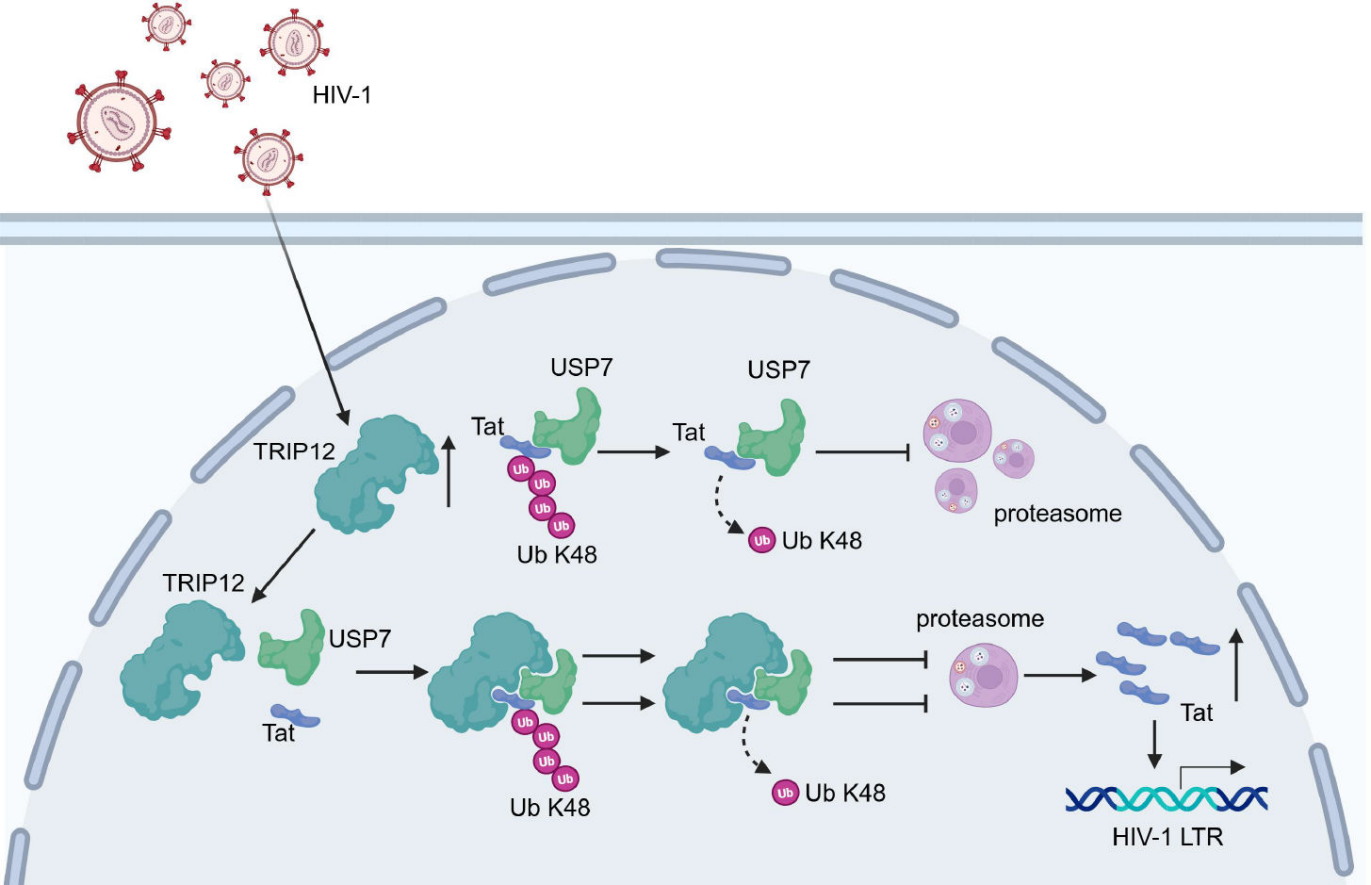
Proposed model for TRIP12-mediated stabilization of Tat via USP7. The schematic illustrates a model in which TRIP12 regulates the stability of the HIV-1 Tat protein. Following HIV-1 infection or latency reactivation, TRIP12 expression is elevated. TRIP12 facilitates the interaction between the deubiquitinase USP7 and Tat, thereby enhancing USP7-mediated deubiquitination of Tat and reducing K48-linked ubiquitination and proteasomal degradation. Consequently, stabilized Tat promotes HIV-1 LTR-driven transcription and viral replication. The schematic model is generated using BioRender (http://biorender.com/).

In summary, this study reveals that TRIP12 prevents proteasomal degradation of the HIV-1 Tat protein by enhancing its interaction with USP7 and reducing K48-linked ubiquitination. Through this mechanism, TRIP12 stabilizes Tat and consequently promotes Tat-dependent viral transcription, replication, and latency reactivation (Fig. 8). These findings provide new insight into the molecular mechanisms governing Tat stability and highlight the pivotal role of the host ubiquitin system in regulating HIV-1 replication and latency maintenance. Notably, although TRIP12 is an HECT-type E3 ubiquitin ligase, our data indicate that its regulation of Tat stability is independent of its intrinsic catalytic activity, suggesting that TRIP12 may function as a molecular scaffold rather than a canonical ubiquitin ligase in the context of Tat regulation. Future studies are warranted to define the specific domains mediating TRIP12-USP7-Tat complex formation and to further elucidate the structural basis of this regulatory axis. In addition, several mechanistic experiments in this study relied on transient overexpression systems in HEK293T cells. Although our data support a role for TRIP12 in regulating Tat protein stability, we cannot fully exclude potential contributions from differences in Tat plasmid transcription, transfection efficiency, or reporter input in these settings. Future studies using more stable expression systems will help further strengthen these conclusions. Nonetheless, our study has certain limitations, as data derived from cultured cells may not fully reflect the physiological role of TRIP12 in humans. Given the central role of Tat in the HIV-1 life cycle, pharmacological modulation of TRIP12, either through activation or inhibition, may represent a promising therapeutic strategy against HIV-1 infection. Overall, our work expands the understanding of host–virus interactions and provides a conceptual framework for targeting host-mediated stabilization of viral regulatory proteins as an antiviral strategy.

## MATERIALS AND METHODS

### Patient and donor samples

Twenty people living with HIV were recruited through the Changchun Center for Disease Control and Prevention (Jilin, China). Among them, eight participants had not received any antiretroviral treatment, whereas 12 individuals had been on highly active antiretroviral therapy (HAART) for more than 3 years and displayed undetectable levels of plasma HIV-1 RNA. Viral loads were measured using a commercially available HIV-1 RNA quantification kit (DaAn Gene Co., DA-Z195). Additionally, peripheral blood CD4+ T cells from nine healthy volunteers were obtained to analyze TRIP12 mRNA expression. CD4+ T cells from three of these healthy donors were subsequently subjected to HIV-1 infection assays following siRNA-mediated TRIP12 depletion. Detailed demographic and clinical data for all participants are provided in Table S1.

### Plasmid construction

The TurboID-Tat expression plasmid used in this study was constructed as previously described in our earlier work (9). The 3× Flag-tagged TRIP12 expression vector was obtained from PPL (Public Protein/Plasmid Library, China). A single nucleotide substitution (C2007A) in TRIP12 was generated by site-directed mutagenesis using the Flag-tagged plasmid as the template. HIV-1-related plasmids, including pNL4-3 and its Env-deficient GFP reporter construct, were obtained from the NIH AIDS Reagent Program.

Tat coding sequence fragments derived from HIV-1 molecular clones (NL4-3, Yu2, AD8, and 89.6) were synthesized by GenScript (Nanjing, China) and subsequently inserted into the VR1012 vector. The LTR-driven luciferase reporter plasmid was constructed by cloning the LTR region of pNL4-3 into the pGL3-Basic vector, and a ΔTAR mutant variant was generated on the same backbone by PCR-based mutagenesis (49). Several HIV-1 infectious molecular clones (IMCs) were kindly provided by Dr. Beatrice Hahn’s laboratory (50–52). Primer sequences used for all plasmid constructions are provided in Table S2.

### Cells and antibodies

Human embryonic kidney 293T (HEK293T; catalog no. CRL-11268), HeLa (catalog no. CCL-2), and TZM-bl (catalog no. PTA-5659) cell lines were obtained from the American Type Culture Collection (ATCC). These cells were cultured in Dulbecco’s modified Eagle medium (DMEM; Thermo Fisher Scientific, catalog no. 11995065) supplemented with 10% fetal bovine serum (FBS; PAN Seratech, catalog no. ST30-3302). The HIV-1 latent C11 cell line was kindly provided by Dr. H. Z. Zhu (College of Life Science, Fudan University). MT-4 (catalog no. 120) and J-Lat 6.3 (catalog no. 9846) cells were supplied by the NIH AIDS Research and Reference Reagent Program. These cell lines, together with Jurkat cells (ATCC, catalog no. TIB-152), were maintained in RPMI 1640 medium supplemented with 10% FBS and Penicillin-Streptomycin solution (Biological Industries, catalog no. 03-031-1B).

Peripheral blood mononuclear cells (PBMCs) were isolated by Ficoll gradient centrifugation, and CD4+ T cells were purified using magnetic microbeads coated with anti-CD4 antibodies (Miltenyi Biotec, catalog no. 130-045-101). All cultures were maintained at 37°C in a humidified incubator containing 5% CO₂.

The following antibodies were used in this study: rabbit anti-HA (Thermo Fisher Scientific, catalog no. 715500), mouse anti-Myc (clone 4A6, Millipore, catalog no. 05-724), mouse anti-Flag (clone M2, Sigma-Aldrich, catalog no. F1804), mouse anti-p24 (ARRRP, catalog no. 1513), mouse anti-β-actin (Genscript, catalog no. A00702-100), rabbit polyclonal anti-TRIP12 (Proteintech, catalog no. 25303-1-AP), mouse monoclonal anti-USP7 (Proteintech, catalog no. 66514-1-Ig), rabbit polyclonal anti-MDM2 (BBI, catalog no. D260611), rabbit polyclonal anti-PARP1 (BBI, catalog no. D261071), HRP-conjugated goat anti-mouse IgG (H + L) (Jackson ImmunoResearch, catalog no. 115-035-062), and HRP-conjugated goat anti-rabbit IgG (H + L) (Jackson ImmunoResearch, catalog no. 111-035-045).

### RNA extraction and RT-qPCR

Total RNA from cultured cells and blood samples was extracted using TRIzol reagent (Invitrogen, 15596018CN) supplemented with an RNase inhibitor and DEPC-treated water. For cDNA synthesis, 1 μg of total RNA was reverse transcribed in a 20 μL reaction using either random hexamer or oligo(dT) primers with a commercial high-capacity cDNA synthesis kit (Roche, 4896866001), following the manufacturer’s instructions.

Quantitative real-time PCR was performed on an Mx3005P instrument using a SYBR Green master mix (Roche, 491314001) containing HotMaster Taq DNA polymerase. Each 12 μL amplification mixture contained 0.5 μL each of forward and reverse primers (10 μM), 2 μL of cDNA template, and the appropriate reaction buffer. The thermal cycling conditions consisted of an initial denaturation at 95°C for 2 min, followed by 40 cycles of 95°C for 15 s, 57°C for 15 s, and 68°C for 20 s. The primer sequences used for quantitative PCR are listed in Table S2.

### Transfection and immunoblot analysis

A total of 3 × 10⁵ adherent cells were plated in 12-well plates and transfected with Lipofectamine 2000 (Invitrogen, 11668-019) according to the manufacturer’s instructions. For siRNA delivery, Lipofectamine RNAiMAX reagent (Invitrogen, catalog no. 13778150) was used following the recommended protocol. Human CD4+ T cells and T-cell lines were transfected by nucleofection using the Amaxa Human T Cell Nucleofector Kit with program U-014 or Kit V with program X-001 (Lonza).

Before cell harvest, cells were treated as required with MG132 (Sigma, C2211), BafA1 (HY-100558, MCE), or the USP7 inhibitor GNE-6640 (HY-112937, MCE) for the indicated durations. Cells were collected 48 h after transfection or stimulation, lysed, and denatured in 1 × loading buffer containing 0.08 M Tris-HCl (pH 6.8), 2% SDS, 10% glycerol, 0.1 M dithiothreitol, and 0.2% bromophenol blue. Protein samples were resolved by SDS-PAGE using 12% polyacrylamide gels and transferred onto nitrocellulose membranes. The membranes were blocked and then incubated with the appropriate primary antibodies diluted in 1% milk prepared in phosphate-buffered saline (PBS), followed by incubation with HRP-conjugated secondary antibodies (Jackson ImmunoResearch). Protein signals were visualized using an enhanced chemiluminescence detection kit (FG Super Sensitive ECL Kit, catalog no. MA0186-1; Meilun Biotechnology Co.).

### siRNA, shRNA, and sgRNA construction

For gene silencing experiments, short interfering RNAs (siRNAs) targeting TRIP12 were synthesized by RiboBio (Guangzhou, China). TRIP12-specific short hairpin RNA (shRNA) sequences were cloned into the lentiviral vector pLKO.1-puro (Addgene, catalog no. 8453). The target sequences were as follows: shTRIP12 forward: 5′-CCGGCCACTACTCAGTCACCTAAATCTCGAGATTAGGTGACTGAGTAGTGGTTTTTG-3′, shTRIP12 reverse: 5′-AATTCAAAAACCACTACTCAGTCACCTAAATCTCGAGATTTAGGTGACTGAGTAGTGG-3′.

The generation of CRISPR-Cas9-mediated TRIP12 and USP7 knockout HEK293T cell lines was performed as described previously (53).

### Construction of stably silenced cell lines

To generate stable knockdown cell lines, HEK293T cells were co-transfected with TRIP12-specific or control pLKO.1 vectors along with the packaging plasmids RRE, REV, and VSV-G using a liposome-based transfection reagent; 48 h after transfection, viral supernatants were harvested and quantified with a Lentiviral p24 Rapid Detection Kit (Jianke, catalog no. JK-009-100, Beijing, China). The collected viral particles were then used to infect T cells, which were subsequently selected with puromycin (3 μg/mL) for 48 h to establish stable cell populations.

### Cell proliferation assay

Cell proliferation was assessed using the CCK-8 colorimetric assay (TransGen Biotech, C6005XL) according to the manufacturer’s instructions. Absorbance at 450 nm was measured at the indicated time points with a microplate reader.

### HIV-1 production, infection, and reactivation

HEK293T cells transfected with the pNL4-3 molecular clone produced HIV-1 particles through standard lipofection. Viral supernatants were collected 48 h post-transfection, and titers were quantified using a p24 ELISA kit (Hebei Medical University, China). T cells were subsequently infected with HIV-1 based on p24 antigen concentration. Specifically, 10 ng of p24 antigen was used to infect 1 × 10⁶ T cells for 4 h at 37°C. After infection, cells were washed three times with phosphate-buffered saline (PBS) to remove unbound virions and then maintained in fresh complete medium. Viral supernatants were harvested at the indicated time points and quantified using the same p24 ELISA kit according to the manufacturer’s protocol. To assess viral infectivity, TZM-bl reporter cells—HeLa derivatives containing an HIV-1 LTR-driven luciferase gene—were seeded in 24-well plates and exposed to the collected viral supernatants. After 48 h, luciferase activity was measured using a luminometer.

For HIV-1 latency reactivation assays, J-Lat 6.3 cells harboring integrated latent proviruses, C11 cells, or primary human CD4+ T cells were either nucleofected with siRNAs targeting TRIP12 or transduced with lentiviruses expressing shTRIP12 to generate stable knockdown cell lines. Following transfection or transduction, cells were stimulated with PMA (20 nM) or PHA-M (5 μg/mL) for the indicated time periods. Viral reactivation was then assessed by flow cytometric (FACSCalibur; BD) analysis of GFP expression and by immunoblotting for p24 protein levels.

### Luciferase assay

HEK293T cells were pre-seeded in 12-well plates and transfected with the indicated plasmids using Lipofectamine 2000 following the manufacturer’s protocol; 48 h post-transfection, cells were harvested for subsequent analysis. Luciferase activity was measured using the Dual-Luciferase Reporter Assay System (catalog no. E1910; Promega), and luminescence was quantified on a GloMax 20/20 luminometer (Promega) according to the manufacturer’s instructions.

### Co-immunoprecipitation (Co-IP)

Transfected HEK293T cells were lysed in PBS containing 1% Triton X-100 and a protease inhibitor cocktail (Roche, 11836170001) at 4°C for 1 h. The lysates were clarified by centrifugation at 10,000 × *g* for 30 min at 4°C, and the resulting supernatants were incubated with agarose beads conjugated to the corresponding antibodies for 4 h under gentle rotation. The beads were subsequently washed six times with wash buffer (20 mM Tris-HCl, pH 7.5, 100 mM NaCl, 0.1 mM EDTA, and 0.05% Tween-20), and bound proteins were eluted and analyzed by IB.

### Identification of host factors interacting with Tat

HEK293T cells were transfected with either TurboID-Tat or TurboID control constructs and maintained for 18–24 h before biotin labeling. Biotin was introduced into the culture medium at a final concentration of 500 μM and allowed to react for 10 min. The labeling process was immediately terminated by transferring the cells to ice, followed by five washes with ice-cold PBS to remove excess biotin. Cells were gently detached by pipetting, collected by centrifugation at 1,500 × *g* for 3 min, and the pellets were resuspended in 1.2 mL of RIPA buffer (50 mM Tris-HCl, pH 8.0, 150 mM NaCl, 0.1% SDS, 0.5% sodium deoxycholate, 1% Triton X-100, 1 × protease inhibitor cocktail, and 1 mM PMSF). Lysis was facilitated by gentle mixing and incubation on ice for 5 min. The lysates were clarified twice by centrifugation at 10,000 × *g* for 10 min each at 4°C.

To isolate biotinylated proteins for proteomic analysis, 50 μL of streptavidin bead slurry was equilibrated with RIPA buffer and incubated overnight at 4°C with clarified lysates under constant rotation. Beads were sequentially rinsed twice with RIPA buffer, once with 1 M KCl, once with 0.1 M Na₂CO₃, once with 2 M urea in 10 mM Tris-HCl (pH 8.0), and again twice with RIPA buffer to ensure high-purity recovery. Bound proteins were released by boiling the beads in 60 μL of 2 × loading buffer supplemented with 20 mM DTT and 2 mM biotin. Mass spectrometric identification was carried out at the core facility of the First Hospital of Jilin University.

### Immunofluorescence assay (IFA)

To examine the subcellular localization of Tat, TRIP12, and USP7, HeLa cells were transfected with expression plasmids for these proteins for 48 h. Cells were fixed with 4% paraformaldehyde at room temperature for 15 min, washed with PBS, permeabilized with 0.1% Triton X-100 for 5 min, and washed again with PBS. After blocking with 2% BSA for 1 h at room temperature, Tat, TRIP12, and USP7 were stained with respective antibodies. Nuclei were counterstained with DAPI (Sigma, D9542). Fluorescence images were acquired using a laser scanning confocal microscope (LSM710, Carl Zeiss).

### Statistical analysis

Statistical tests are specified in the corresponding figure legends. The results are shown as means ± SDs. Statistical significance was defined as: *P* < 0.05 (*), *P* < 0.01 (**), *P* < 0.001 (***), and *P* < 0.0001 (****). Differences with *P* < 0.05 were considered statistically significant. “ns” denotes no significant difference.

## Data Availability

The authors declare that all data supporting the results of this study can be obtained in the paper and its supplemental material.

## SUPPLEMENTAL MATERIAL

Supplemental material jvi.00396-26-s0001.docx Fig. S1 to S5; Tables S1 and S2.

ASM does not own the copyrights to Supplemental Material that may be linked to, or accessed through, an article. The authors have granted ASM a non-exclusive, world-wide license to publish the Supplemental Material files. Please contact the corresponding author directly for reuse.
