# Supplementary material for: TRIP12 promotes HIV-1 replication and latency reactivation by stabilizing Tat via USP7-mediated deubiquitination
Source: J Virol. 2026 May 13;100(6):e00396-26. doi: 10.1128/jvi.00396-26 (PMC13288937; doi:10.1128/jvi.00396-26)
Supplement: Supplemental material — Fig. S1 to S5; Tables S1 and S2. [file jvi.00396-26-s0001.docx]

Supplemental Material For
TRIP12 promotes HIV-1 replication and latency reactivation by stabilizing Tat via USP7-mediated deubiquitination

Hongyun Shi^1,2^, Panpan Quan^3^, Yubao Hou^1^, Huihan Wang^1^, Yingchao Wang^2^, Hong Wang^1^* and Wenyan Zhang^1^*

^1^Institute of Virology and AIDS Research, Center of Infectious Diseases and Pathogen Biology, Key Laboratory of Organ Regeneration and Transplantation of the Ministry of Education, the First Hospital of Jilin University, Changchun, China;

^2^Hepatobiliary Pancreatic Surgery, the First Hospital of Jilin University, Changchun, China

^3^Department of Cadre's Wards Ultrasound Diagnostics, Ultrasound Diagnostic Center, The First Hospital of Jilin University, Changchun, Jilin, China;

*Corresponding author

Wenyan Zhang (zhangwenyan@jlu.edu.cn), Hong Wang (wanghong_2020@jlu.edu.cn)

Competing Interests: The authors declare no conflicts of interest.


This PDF file includes:
Supplemental Fig S1-5
Supplemental Table S1-2

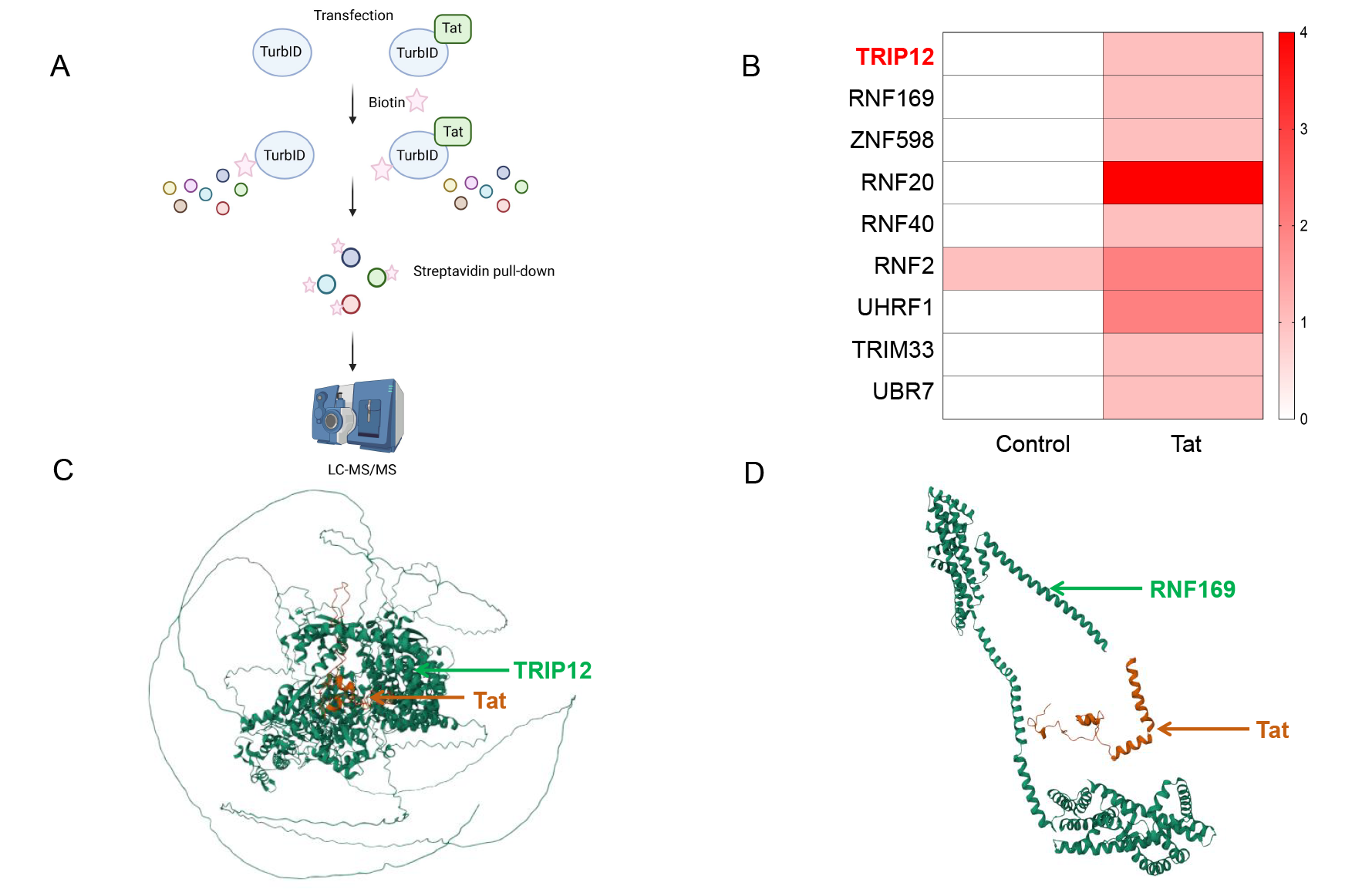

**Fig S1 Identification of the E3 ubiquitin ligase TRIP12 as a Tat-associated factor by TurboID-based proximity labeling.**
(A) Schematic diagram showing the experimental workflow of TurboID-based proximity labeling. HEK293T cells were transfected with TurboID or TurboID-Tat plasmids, followed by biotin labeling and streptavidin pull-down. The biotinylated proteins were subjected to LC-MS/MS analysis to identify potential Tat-interacting proteins. (B) Heatmap showing E3 ubiquitin ligases upregulated in the Tat group compared with the control. (C-D) Predicted 3D interaction models of Tat with TRIP12 (C) and RNF169 (D) generated using AlphaFold3. The schematic in (A) is generated using BioRender (http://biorender.com/).


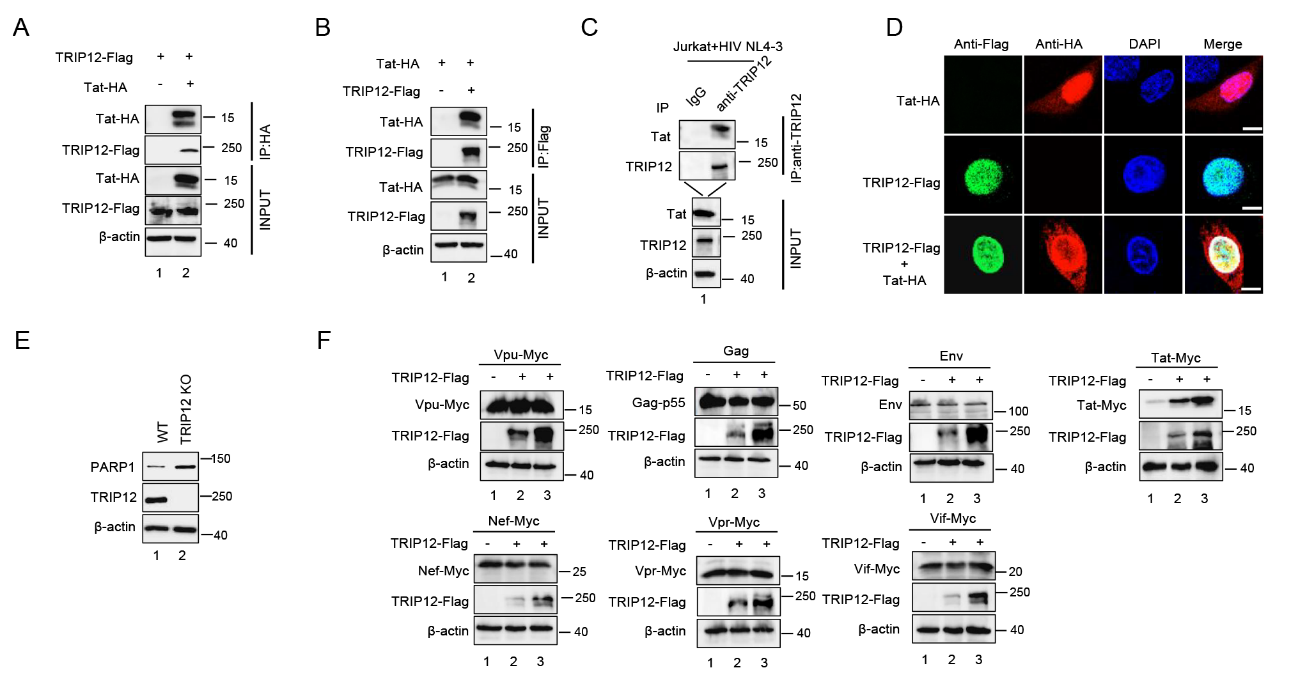

**Fig S2 TRIP12 interacts with Tat.**
(A-B) Co-IP assays showing the interaction between TRIP12 and Tat. HEK293T cells were co-transfected with plasmids expressing HA-tagged Tat and Flag-tagged TRIP12 as indicated. Cell lysates were immunoprecipitated with anti-HA (A) or anti-Flag (B) antibodies, followed by IB with the indicated antibodies. (C) Endogenous association between TRIP12 and Tat in HIV-1-infected Jurkat cells. Jurkat cells were infected with HIV-1 NL4-3 generated from transfected HEK293T cells. 48 h post-infection, cell lysates were subjected to co-IP using an anti-TRIP12 antibody, followed by IB for Tat. Normal IgG served as a negative control. (D) Confocal microscopy showing the subcellular co-localization of TRIP12-Flag (green) and Tat-HA (red) in HEK293T cells. Nuclei were stained with DAPI (blue). Scale bars, 10 μm. (E) Functional validation of TRIP12 knockout. WT and TRIP12 KO HEK293T cells were analyzed by IB using the indicated antibodies. PARP1, a reported endogenous target of TRIP12, was increased in TRIP12 KO cells. (F) Effect of TRIP12 on the expression of HIV-1 proteins. HEK293T cells were co-transfected with TRIP12-Flag and Myc-tagged HIV-1 proteins (Vpu, Gag, Env, Nef, Vpr, Vif, or Tat). Cell lysates were analyzed by IB using the indicated antibodies. TRIP12 preferentially enhanced Tat protein abundance, whereas no obvious changes were observed for other viral proteins under the same conditions. Prior to cell harvest, cells were treated with 10 µM MG132 for 8 h to ensure that Tat protein levels were comparable across all samples. This approach allowed accurate assessment of the interactions between target factors in subsequent co-IP experiments without interference from variations in input levels (A and B).


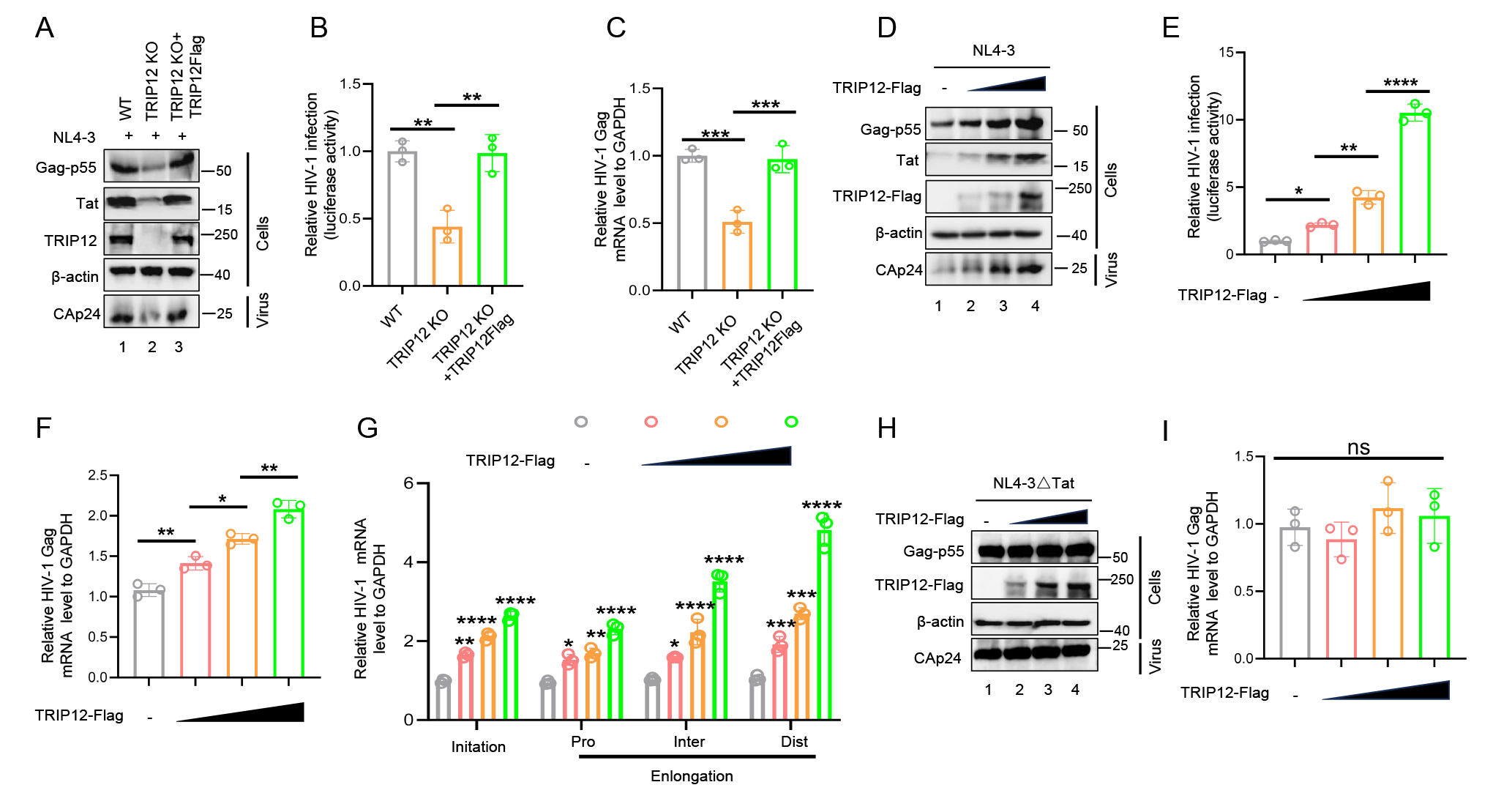


Fig S3 TRIP12 promotes HIV-1 replication and transcription in HEK293T cells.

(A-C) TRIP12 knockout reduces HIV-1 production, while re-expression of TRIP12 restores viral output. HEK293T cells were transfected with pNL4-3 and the indicated TRIP12 constructs. Cell lysates were analyzed by IB (A), and infectious virus release was quantified in TZM-bl cells (B). HIV-1 Gag mRNA levels were determined by RT-qPCR (C). (D-G) TRIP12 enhances infectious HIV-1 production in a dose-dependent manner. HEK293T cells were transfected with pNL4-3 together with increasing amounts of TRIP12-Flag plasmid. At 48 h post-transfection, cell lysates and supernatants were collected for IB analysis of Gag-p55, Tat and CAp24 levels (D). Infectious virus production was quantified in TZM-bl reporter cells (E), and HIV-1 Gag mRNA levels were measured by RT-qPCR (F). (G) RT-qPCR analysis of HIV-1 transcripts corresponding to different transcriptional stages, including initiation, promoter-proximal, intermediate, and distal elongation, normalized to GAPDH. (H-I) TRIP12 fails to promote HIV-1 replication and transcription in the absence of Tat. HEK293T cells were co-transfected with increasing amounts of TRIP12 and a Tat-deficient HIV-1 clone (pNL4-3ΔTat). Cell lysates and supernatants were analyzed by IB to detect Gag-p55 and CAp24 levels (H), and Gag mRNA levels were measured by RT-qPCR (I). Data are shown as means ± SDs from three independent experiments. *P* values were calculated using one-way ANOVA (B, C, E, F, and I) or two-way ANOVA (G). Significance: **P* < 0.05, ***P* < 0.01, ****P* < 0.001, *****P* < 0.0001, ns (not significant).


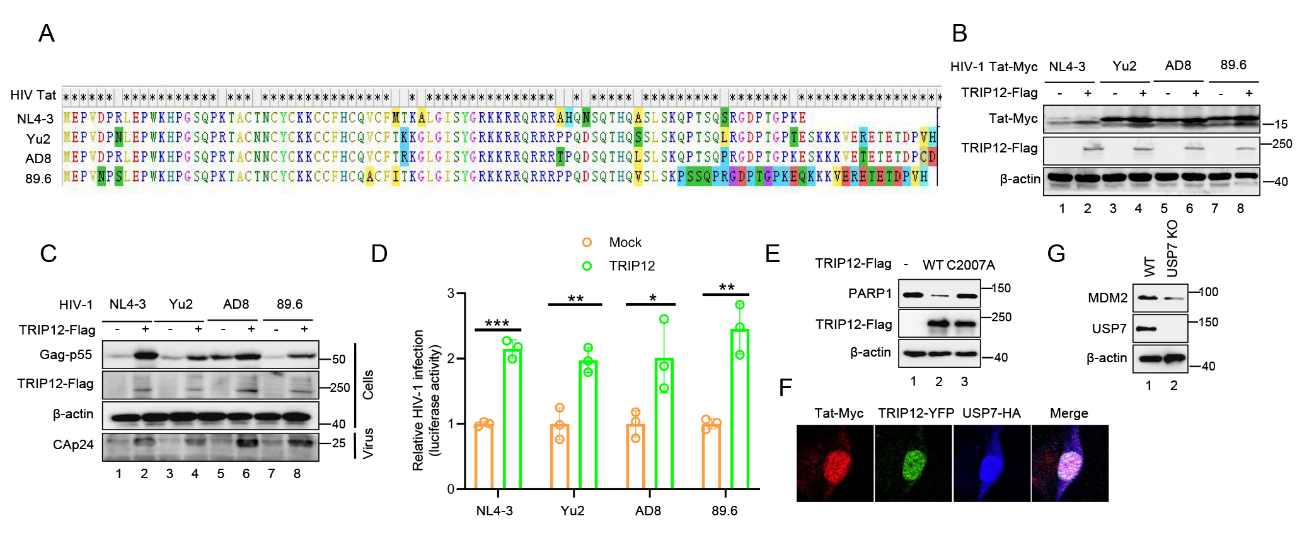

Fig S4 TRIP12 enhances Tat stability and promotes replication of HIV-1 strains with different coreceptor tropisms.
(A) Amino acid sequence alignment of HIV-1 Tat proteins derived from NL4-3, Yu2, AD8, and 89.6 strains. Sequence alignment was performed using Clustal Omega. (B) TRIP12 stabilizes Tat proteins derived from multiple HIV-1 strains. HEK293T cells were co-transfected with TRIP12-Flag and Myc-tagged Tat plasmids corresponding to HIV-1 (NL4-3, Yu2, AD8, 89.6). After 48 h, cell lysates were analyzed by IB using the indicated antibodies. (C) TRIP12 enhances viral protein expression across HIV-1 strains with distinct coreceptor usages, including Yu2 and AD8 (CCR5-tropic), 89.6 (R5X4-tropic), and NL4-3 (CXCR4-tropic). HEK293T cells were co-transfected with TRIP12-Flag and the corresponding HIV-1 molecular clones, followed by IB analysis of Gag-p55 and CAp24 at 48 h post-transfection. (D) Infectious virus yield was quantified in TZM-bl reporter cells by measuring luciferase activity from supernatants of (C). (E) Functional validation of the TRIP12 C2007A mutant. HEK293T cells were transfected with empty vector, wild-type TRIP12-Flag, or TRIP12 C2007A-Flag plasmids as indicated. Cell lysates were analyzed by IB using the indicated antibodies. Wild-type TRIP12, but not the C2007A mutant, reduced PARP1 abundance. (F) Confocal microscopy of transfected HeLa cells showed nuclear colocalization of Tat (red), TRIP12 (green), and USP7 (blue). Scale bar, 10 μm. (G) Functional validation of USP7 knockout. WT and USP7 KO HEK293T cells were analyzed by IB using the indicated antibodies. MDM2, a well-established endogenous target stabilized by USP7, was reduced in USP7 KO cells. Data are shown as means ± SDs from three independent experiments. *P* values were calculated using two-tailed unpaired Student's *t*-test. Significance: **P* < 0.05, ***P* < 0.01, ****P* < 0.001.


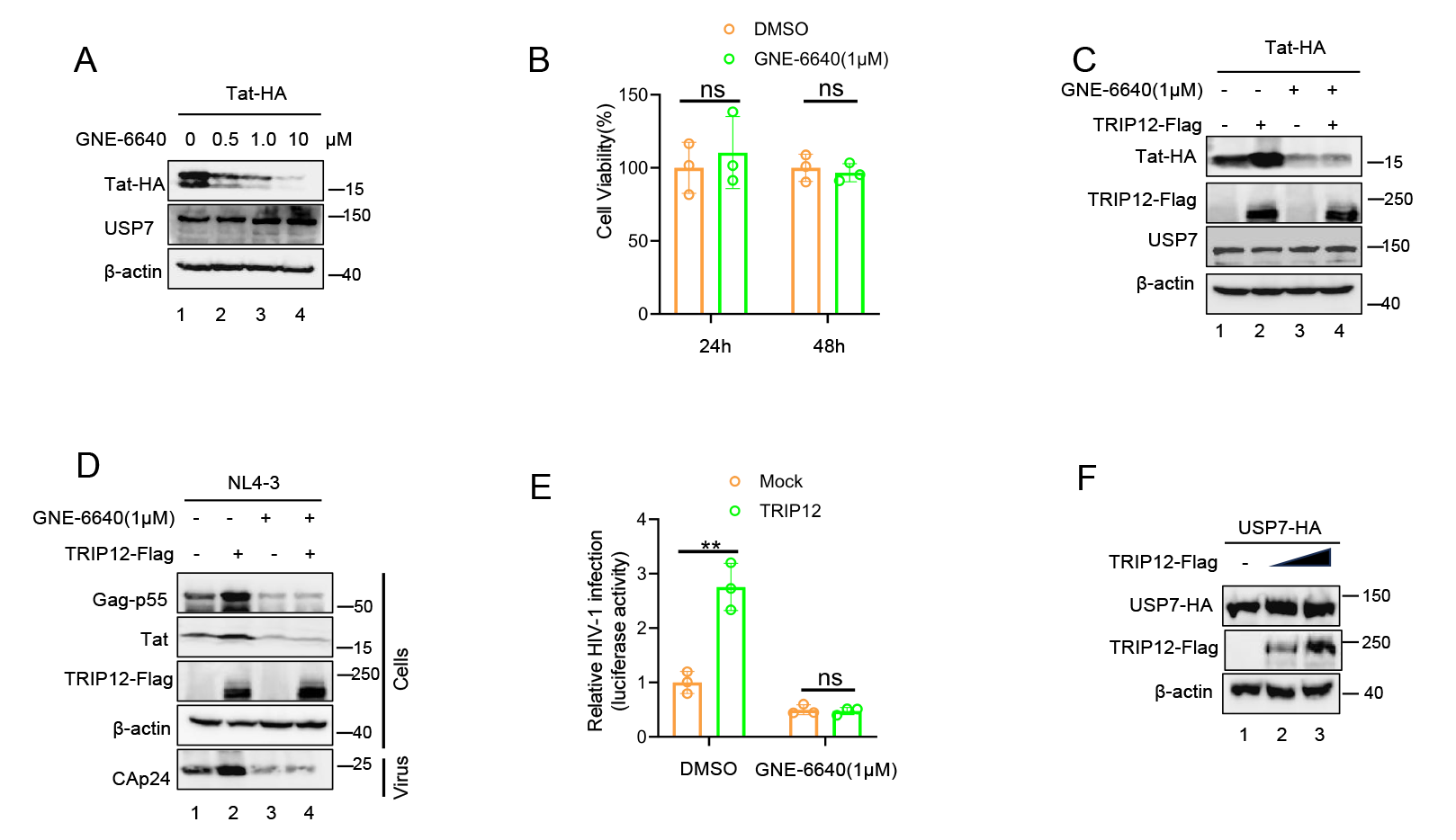

Fig S5 USP7 inhibition attenuates the ability of TRIP12 to stabilize Tat and promote HIV-1 replication.
(A) Treatment with the USP7 inhibitor GNE-6640 decreased Tat protein levels in a dose-dependent manner. HEK293T cells expressing Tat-HA were treated with increasing concentrations of the USP7 inhibitor GNE-6640 (0-10 μM) for 24 h, beginning at 24 h post-transfection, followed by IB analysis with the indicated antibodies. (B) Cell viability was measured after treatment with 1 μM GNE-6640 for 24 h and 48 h using the CCK-8 assay, showing no cytotoxicity at this concentration. (C-E) Pharmacological inhibition of USP7 attenuates TRIP12-mediated stabilization of Tat and enhancement of HIV-1 replication. HEK293T cells were co-transfected with TRIP12-Flag and Tat-HA (C) or pNL4-3 (D) and treated with GNE-6640 (1 μM, 24 h). Tat and Gag-p55 in cells and CAp24 in supernatants were detected by IB (D). Infectious HIV-1 production was quantified in TZM-bl cells using a luciferase activity assay (E). (F) TRIP12 does not affect USP7 stability. HEK293T cells were co-transfected with USP7-HA and increasing amounts of TRIP12-Flag plasmids, and cell lysates were analyzed by IB. Consistent with previous reports suggesting that USP7 may influence TRIP12 stability, TRIP12 expression was carefully monitored and adjusted to comparable levels across experimental conditions to ensure accurate assessment of its effect on Tat (C and D). Data are shown as means ± SDs from three independent experiments. *P* values were calculated using two-tailed unpaired Student's *t*-test (B and E). Significance: ns (not significant).

S1 Table. Characteristics of people living with HIV in this study

| Characteristic^a^ | Not treated with HAART^b^ | Treated with HAART |
| --- | --- | --- |
| No. of PLWH | 8 | 12 |
| Sex | 3 females, 5 males | 12 males |
| Age (yrs) | 39.4 ± 13.5 | 40.8 ±9.1 |
| Mode of infection | Sexual transmission | Sexual transmission |
| Viral load (IU/mL) | 1.9E5 ± 1.3E5 | ＜50 |
| CD4+ T count (cells/μL) | 356 ± 55 | 998 ± 105 |
| Antiretroviral therapy | No | Yes |
| Duration of antiretroviral therapy (yrs) | No | 3.6 ± 1.7 |
| Treatment regimen | No | Tenofovir disoproxil +  lamivudine + efavirenz |

a All values are means ± SDs if not indicated otherwise.

b Participants in this group were newly diagnosed people living with HIV who had not received HAART.

**S2 Table. Primers used for plasmid construction and RT-qPCR.**

| Primer name | Primer direction | Sequence (5’-3’) |
| --- | --- | --- |
| Flag-TRIP12-C2007A-F | Forward | CCCTCTGTAATGACTGCTGTGAACTATCTTAAGTT |
| Flag-TRIP12-C2007A-R | Reverse | AAGTCATTACAGAGGGCAAGAAGTC |
| GAPDH-RT-F | Forward | TGCACCACCAACTGCTTAGC |
| GAPDH-RT-R | Reverse | GGCATGGACTGTGGTCATGAG |
| TRIP12-RT-F | Forward | ATGTCCAACCGGCCTAATAACA |
| TRIP12-RT-R | Reverse | TCCTATTGAGTCGTCTTGTGGT |
| HIV-Gag-RT-F | Forward | GTGTGGAAAATCTCTAGCAGTGG |
| HIV-Gag-RT-R | Reverse | CGCTCTCGCACCCATCTC |
| Initial-RT-F (Target 10-59 bp) | Forward | GTTAGACCAGATCTGAGCCT |
| Initial-RT-R (Target 10-59 bp) | Reverse | GTGGGTTCCCTAGTTAGCCA |
| Proximal-RT-F (Target 29-180 bp) | Forward | TGGGAGCTCTCTGGCTAACT |
| Proximal-RT-R (Target 29-180 bp) | Reverse | TGCTAGAGATTTTCCACACTGA |
| Intermediate-RT-F (Target 836-1015 bp) | Forward | GTAATACCCATGTTTTCAGCATTATC |
| Intermediate-RT-R (Target 836-1015 bp) | Reverse | TCTGGCCTGGTGCAATAGG |
| Distal-RT-F (Target 2341-2433 bp) | Forward | GAGAACTCAAGATTTCTGGGAAG |
| Distal-RT-R (Target 2341-2433 bp) | Reverse | AAAATATGCATCGCCCACAT |
